# Recent Advances in Small-Molecule-Based Nanovectors for the Treatment of Colorectal Cancer

**DOI:** 10.3390/molecules31193534

**Published:** 2026-10-03

**Authors:** Cuthbert Wasswa Kibungu, Nkoana Ishmael Mongalo, Tshepiso Jan Makhafola

**Affiliations:** 1 Department of Health Sciences, Faculty of Health and Environmental Sciences, Central University of Technology, Bloemfontein 9301, Free State, South Africa; 2 Centre for Quality of Health and Living, Faculty of Health and Environmental Sciences, Central University of Technology, Bloemfontein 9301, Free State, South Africa; 3 College of Agriculture and Environmental Sciences Laboratories, University of South Africa, Private Bag X06, Florida 0610, Gauteng, South Africa; ishmaelmongalo@gmail.com; 4 Stellenbosch Institute for Advanced Study (STIAS), Wallenberg Research Centre at Stellenbosch University, Stellenbosch 7600, Western Cape, South Africa; 5 Hamburg Institute for Advanced Study (HIAS), Rothenbaumchaussee 45, 20148 Hamburg, Germany

**Keywords:** colorectal cancer, nanotechnology, nanovectors, anticancer, drug delivery system, natural products, bioactive compounds

## Abstract

Colorectal cancer (CRC) remains a major global health burden, while conventional systemic therapies are limited by toxicity, drug resistance, suboptimal tumour exposure, and pharmacokinetic constraints. Recent advances in nanotechnology have produced diverse nanosystems including liposomes, dendrimers, polymeric nanoparticles, metallic nanoparticles, mesoporous silica systems, and other hybrid platforms that can improve drug solubility, stability, biodistribution, controlled release, and tumour-directed delivery. Plant-derived small molecules provide an additional source of structurally diverse anticancer candidates, but their clinical development is often constrained by poor aqueous solubility, rapid metabolism, limited bioavailability, and non-specific toxicity. This review critically examines recent progress in nanoscale delivery systems for CRC and evaluates their potential for delivering plant-derived and other bioactive small molecules. Particular emphasis is placed on passive and active targeting, physicochemical determinants of delivery, platform-specific advantages and limitations, preclinical evidence, and the barriers to clinical translation. The review also highlights the distinction between plant-derived and fungal-derived natural products and identifies opportunities for integrating natural products with responsive and ligand-functionalized nanocarriers.

## 1. Introduction

Colorectal cancer (CRC) remains a major cause of cancer morbidity and mortality worldwide. Recent epidemiological evidence identifies CRC as the third most diagnosed cancer and the second leading cause of cancer-related death, while also highlighting important regional differences in incidence and risk [1,2]. Recent global cancer-burden evidence further supports the continuing importance of modifiable risk factors in cancer prevention. Fatalities due to colorectal cancer are not limited to age or a country’s developmental status. It is estimated that over 650,000 people worldwide succumb to this type of cancer annually [3]. CRC generally develops through a multistep process involving precursor lesions and progressive molecular and cellular changes. Early detection and stage-appropriate management remain important determinants of outcome. Current management may involve surgery, chemotherapy, radiotherapy, targeted therapy, and where appropriate, immunotherapy, with treatment selected according to disease stage, tumour biology, and patient characteristics [4,5,6]. Despite therapeutic advances, systemic treatment can be limited by toxicity, resistance, inadequate drug exposure, and difficulties in selectively delivering therapeutic concentrations to tumour tissue [5,6,7].

**Figure 1:**
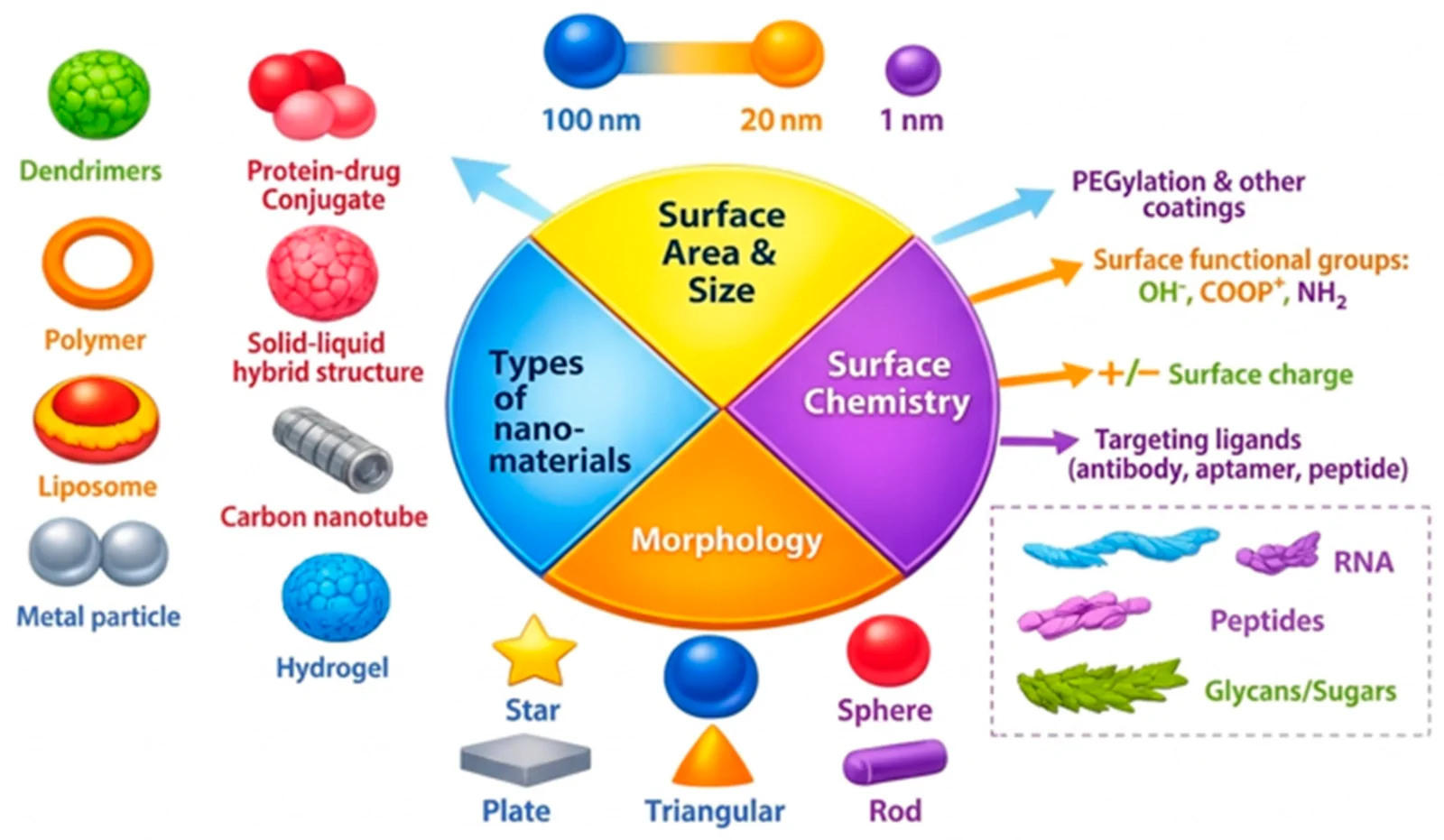
Morphology, surface area, surface modification, and morphology of the nanomaterials used in cancer treatment.

Advances in nanotechnology have created opportunities to address several of these limitations by engineering nanoscale carriers with defined physicochemical properties and targeting functions [8]. Nanovectors can incorporate therapeutic or diagnostic agents and can be designed to modify solubility, circulation time, biodistribution, cellular uptake, and drug-release behaviour [9,10,11,12]. Major platforms include liposomes, polymeric nanoparticles, dendrimers, micelles, metallic nanoparticles, mesoporous silica systems, and carbon-based materials, as seen in Figure 1, as well as other hybrid nanosystems. Their performance depends on particle size, surface characteristics, composition, drug–carrier interactions, and the biological environment encountered after administration [13,14,15,16].

Recent work has increasingly moved beyond simple drug encapsulation towards multifunctional systems that combine controlled release, tumour-responsive behaviour, active targeting, imaging, and combination therapy [17]. Nevertheless, improved laboratory performance does not automatically translate into clinical benefit; formulation reproducibility, toxicity, clearance, manufacturing scale-up, pharmacokinetics, and regulatory requirements remain important barriers [14,16,18]. Accordingly, this review focuses on recent developments while critically comparing the principal nanocarrier platforms and their suitability for colorectal cancer applications.

The review therefore examines conventional treatment limitations, passive and active targeting, major nanocarrier platforms, preclinical CRC applications, and the integration of natural products with nanodelivery systems [19,20]. Particular attention is given to recent literature and to the advantages, limitations, and translational prospects of each approach.

The nanotechnology literature has progressed from relatively simple encapsulation and circulation-extension strategies in the early 2010s toward ligand-functionalised, stimuli-responsive, multimodal, and increasingly biomimetic systems by 2021–2026 [11,14,15,16,21,22,23,24,25,26,27,28,29,30,31,32,33,34,35,36,37,38,39]. Importantly, this progression should not be interpreted as a linear replacement of older platforms. Liposomes, polymeric nanoparticles, and chitosan systems remain relevant because they provide comparatively understandable formulation and release behaviour, whereas newer architectures add functionality at the cost of greater manufacturing, characterisation, and regulatory complexity. For natural products, the central question is therefore not whether the newest nanocarrier is intrinsically superior, but whether its added functionality solves a defined limitation of the selected molecule, such as poor solubility, rapid metabolism, inadequate tumour exposure, or resistance to cellular uptake. This payload-platform matching principle is particularly relevant to compounds discussed later, including berberine, nitidine chloride, solasonin, berbamine, the Melodinus-derived alkaloids, and the fungal metabolite Chaetocochin J.

## 2. Conventional Treatment Approaches for Colorectal Cancer

For over six decades, 5-fluorouracil (5-FU), a fluoropyrimidine-based chemotherapeutic agent, has remained one of the most widely used drugs in cancer treatment, including for breast, colorectal, brain, and thoracic malignancies [7]. When used in combination, these drugs exert multiple cytotoxic effects, notably by inhibiting thymidylate synthase, an effect associated with improved survival in patients with stage III colorectal cancer [4]. 5-FU is a water-soluble antineoplastic compound typically administered intravenously. Other commonly used agents in colorectal cancer (CRC) therapy include irinotecan, capecitabine, oxaliplatin, bevacizumab, panitumumab, and cetuximab. In standard clinical practice, 5-FU is often combined with leucovorin for the treatment of stage II CRC. An enhanced regimen incorporating 5-FU, leucovorin, and oxaliplatin has demonstrated superior efficacy compared with the dual combination of 5-FU and leucovorin alone. Capecitabine serves as an oral alternative to 5-FU, which requires intravenous administration [40]. Although therapeutically beneficial to various forms of cancer, these agents are frequently associated with significant toxicity, leading to adverse effects in the gastrointestinal, nervous, skin, and cardiovascular systems, and may also contribute to the development of chemoresistance [5].

**Figure 2:**
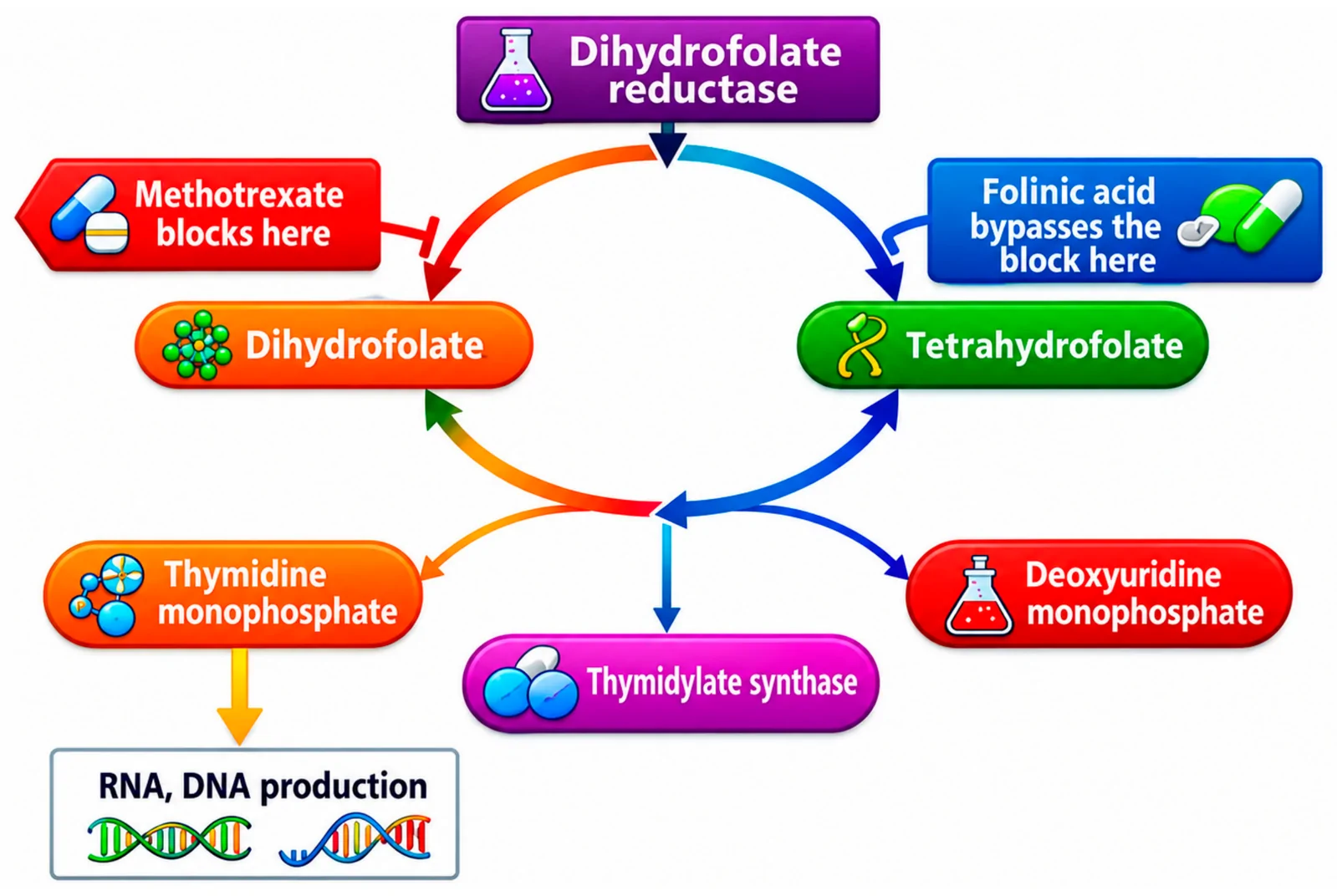
Flowchart showing the mechanism of action of conventional anticancer drugs.

Mechanistically, 5-FU acts by irreversibly inhibiting thymidylate synthase via its active metabolite fluorodeoxyuridine monophosphate (FdUMP), thereby disrupting the production of thymidine, a critical nucleoside required for DNA replication. Leucovorin (folinic acid) is co-administered with 5-FU because it stabilises the binding of the FdUMP–thymidylate synthase complex, thereby enhancing the drug’s anticancer activity. Additional 5-FU metabolites, including fluorouridine triphosphate (FUTP) and fluorodeoxyuridine triphosphate (FdUTP), can be incorporated into RNA and DNA, respectively, resulting in nucleic acid damage. Irinotecan (Camptosar^®^) is a camptothecin-derived prodrug that is converted into an active topoisomerase I inhibitor, inducing DNA damage that ultimately triggers apoptosis. In recent years, agents targeting the epidermal growth factor (EGF) and vascular endothelial growth factor (VEGF) signalling pathways have become increasingly important in managing metastatic CRC, particularly because their anti-angiogenic properties have contributed to improved patient outcomes. Oxaliplatin (Eloxatin^®)^, a third-generation platinum-based drug, undergoes non-enzymatic biotransformation to generate reactive intermediates that form both intra- and inter-strand DNA crosslinks. Figure 2 illustrates the mechanism of action of most anticancer drugs.

These lesions disrupt DNA replication and transcription, ultimately leading to cell death. In addition, its toxicity is partly associated with reactive oxygen species (ROS) generation, which can damage lipids, proteins, polypeptides, and DNA [5]. Advancements in targeted therapy have significantly improved survival rates in CRC patients, contributing to a notable reduction in mortality [6]. The integration of such therapeutic agents with nanosystems (NSs) presents a promising avenue for developing more precise, site-specific treatment strategies. This approach may help minimize systemic toxicity associated with conventional chemotherapeutics while enhancing drug bioavailability and increasing accumulation within the tumor tissues, thereby improving overall therapeutic efficacy.

Chemotherapy involves the use of anticancer agents to eradicate cancer. These drugs are designed to destroy cancerous cells, prevent their spread, or inhibit their growth. Despite these promising benefits, the overall therapeutic process has disadvantages. One major disadvantage is that these drugs cannot distinguish between healthy and cancerous cells. This may well lead to the destruction of healthy cells during chemotherapy, resulting in organ dysfunction and failure, myelosuppression, alopecia, and mucositis [41]. Another major drawback is that these drugs remain in the body for a short time, thereby not interacting optimally with cancerous cells. These chemotherapeutic drugs have poor solubility, limiting their ability to penetrate biological membranes [42].

**Figure 3:**
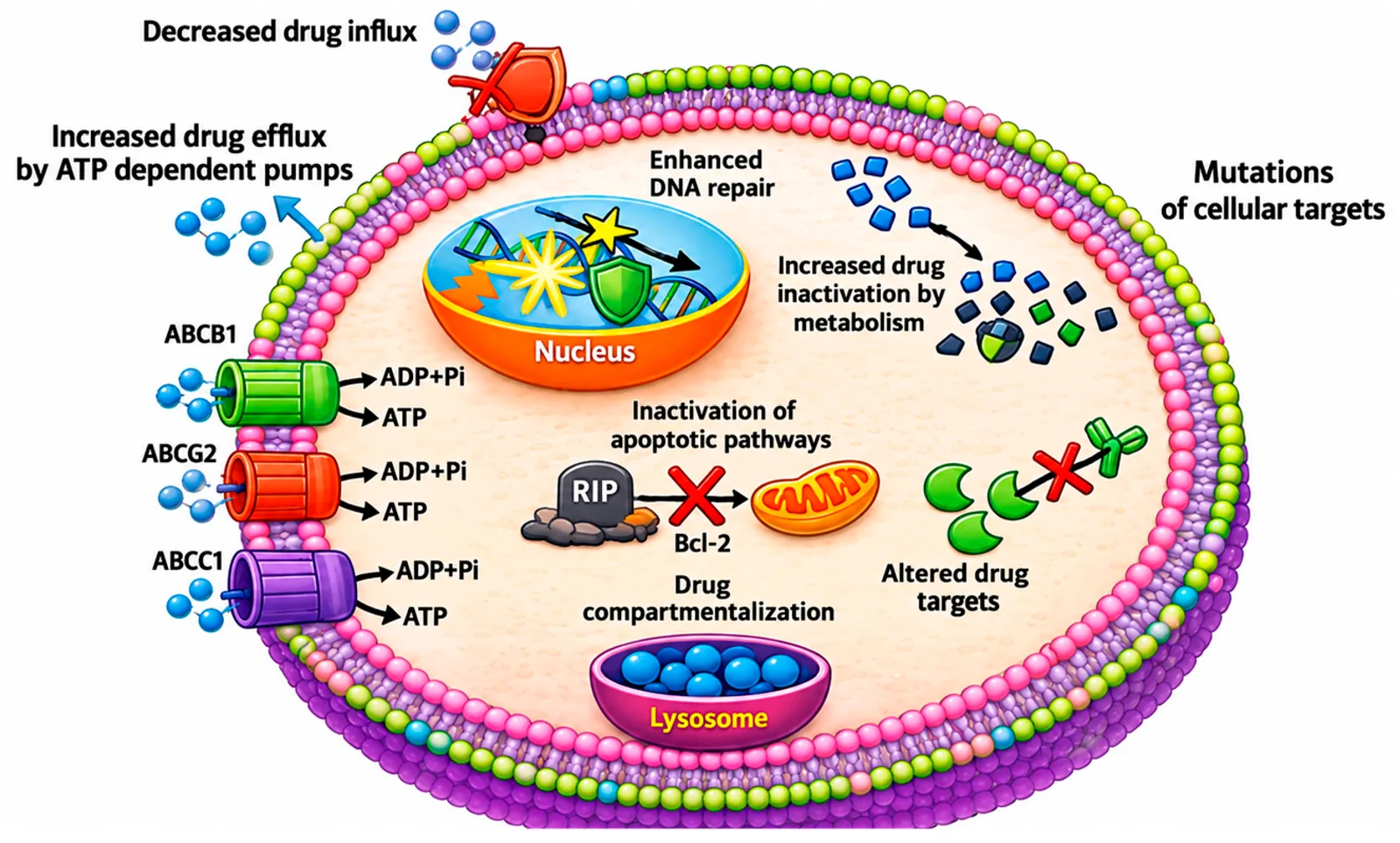
Anticancer drug resistance mechanisms in tumour cells.

Drug resistance may arise through mechanisms operating at the tumour-cell surface and intracellularly, including drug-efflux transporters such as P-glycoprotein, altered drug targets, enhanced DNA repair, and changes in drug metabolism, as illustrated in Figure 3. These mechanisms can reduce intracellular drug exposure and contribute to treatment failure [7,41]. Nanocarrier systems are therefore being investigated as one strategy for improving intracellular delivery and overcoming selected resistance mechanisms. The principal advantages and limitations of several nanocarrier classes are summarised in Table 1 [13,21,43,44,45,46,47].

### 2.1. Passive Targeting Approaches

**Figure 4:**
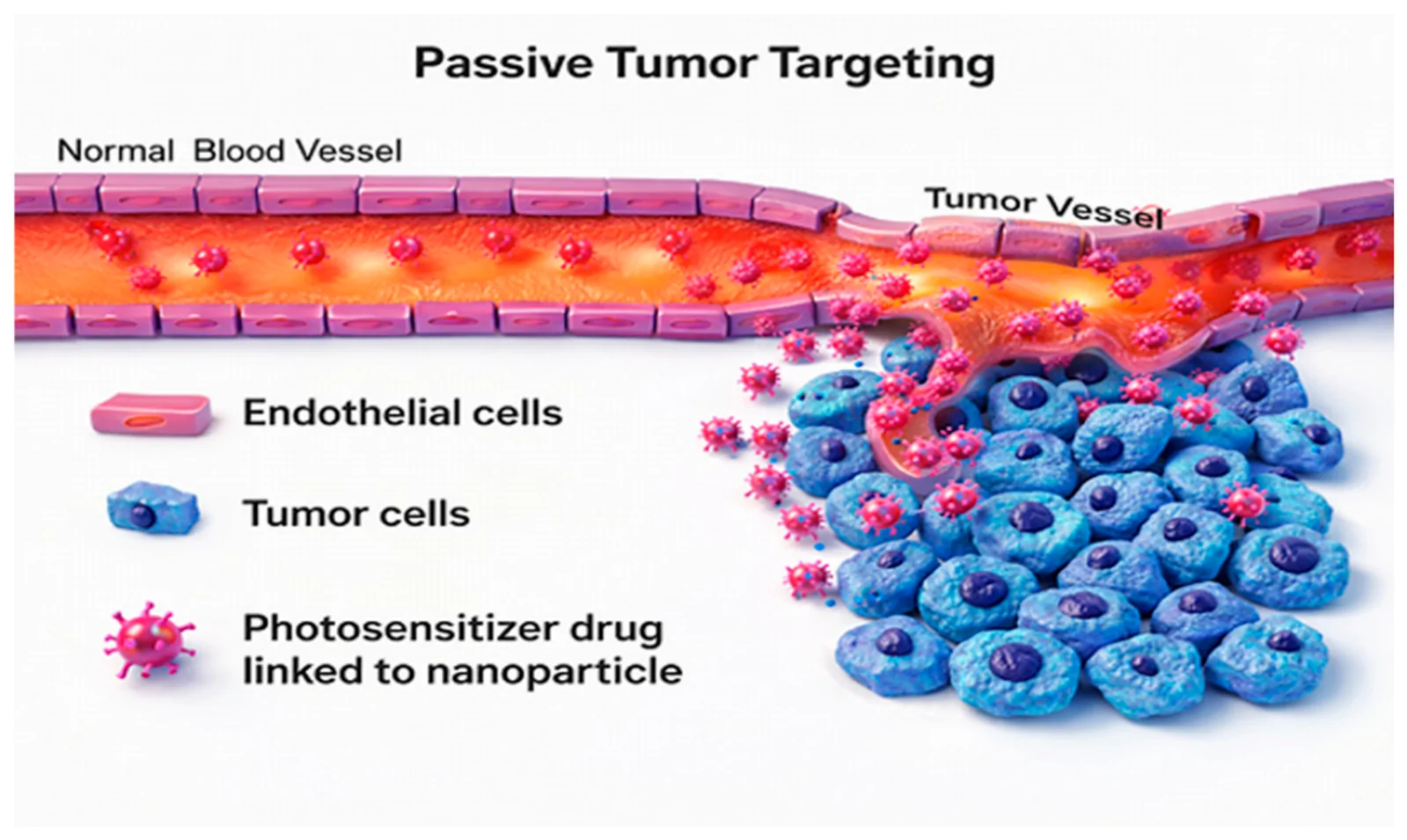
Passive targeting of nanodrug delivery systems.

Passive targeting exploits differences between tumour and normal tissue, particularly abnormal tumour vasculature, impaired lymphatic drainage, and prolonged circulation of appropriately engineered nanosystems. Figure 4 illustrates the passive targeting of tumour cells. The enhanced permeability and retention (EPR) effect can contribute to tumour accumulation, although its magnitude varies substantially between tumour models and patients. Consequently, passive accumulation should not be regarded as a universal or sufficient targeting mechanism. Particle size, surface charge, circulation time, protein adsorption, and clearance by the mononuclear phagocyte system all influence delivery efficiency [11,12,14,15,48,49].

Recent approaches increasingly combine passive accumulation with stimuli-responsive release, including pH-, redox-, enzyme-, temperature-, light-, or other externally triggered mechanisms. These approaches are intended to reduce premature drug release and increase the concentration of active drug at the intended site [11,12,16,50]. The detailed performance of individual carrier platforms is discussed in Section 3.

From 2010 to 2026, the field has increasingly recognised that EPR-mediated accumulation is heterogeneous and often less predictable in human tumours than in small-animal models [11,15,50,51,52]. Consequently, passive targeting is better viewed as a distributional opportunity than a tumour-specific mechanism. For plant-derived payloads, this distinction matters because improved accumulation of a poorly soluble compound such as berberine or curcumin is only useful if the formulation also provides adequate release, intracellular availability, and tolerability. Future studies should therefore report tumour-to-normal-tissue exposure, free-drug release, pharmacokinetics, and pharmacodynamics rather than relying on nanoparticle accumulation or cell viability alone [14,16,35,36,37,38,39,53].

### 2.2. Active Targeting Approaches

**Figure 5:**
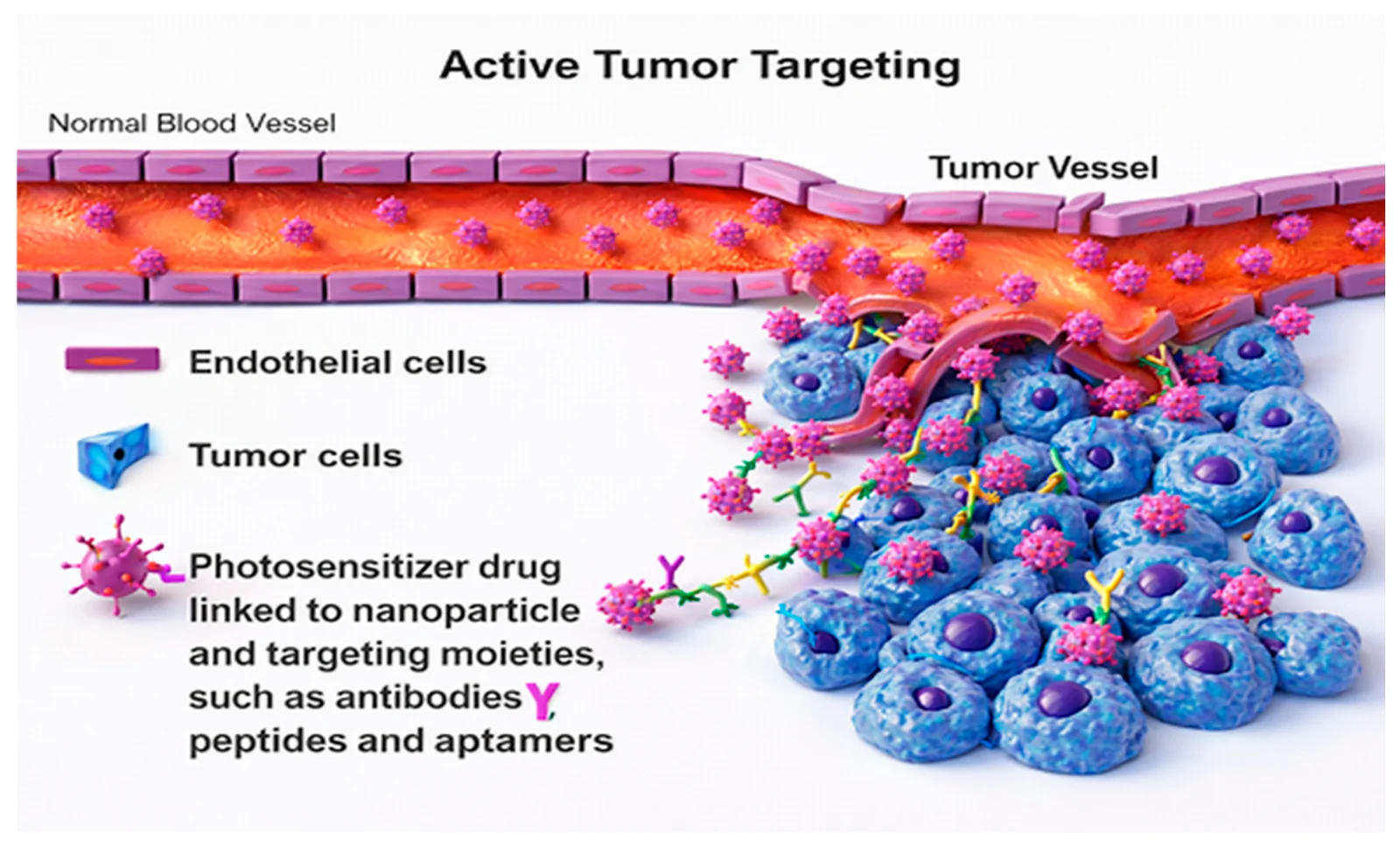
Active targeting of nanodrug delivery systems.

Active targeting adds a recognition component to the nanocarrier surface, such as an antibody, antibody fragment, peptide, aptamer, sugar, or other ligand, to promote interactions with receptors or biomarkers associated with tumour cells or the tumour microenvironment. This strategy can enhance cellular uptake and intracellular delivery, but ligand binding does not eliminate the biological barriers imposed by circulation, protein corona formation, tumour penetration, endosomal trafficking, and clearance. Figure 5 illustrates how active targeting is approached [14,15,16,50,54].

The biggest advantage of active targeting is therefore not simply greater tumour accumulation, but the potential to alter cellular distribution after the carrier reaches the tumour. Recent ligand-functionalized systems illustrate how active targeting can be combined with controlled release and multimodal functions; however, receptor heterogeneity and off-target binding remain important limitations [15,16,24,25,26,27]. Platform-specific examples are considered in Section 3, and integration strategies are discussed in Section 7. The major advance from early ligand-targeted systems to 2026 is a shift from the assumption that receptor binding automatically produces therapeutic selectivity toward recognition that receptor heterogeneity, ligand density, protein-corona formation, endosomal trafficking, and tumour penetration can all weaken targeting performance [15,16,24,25,26,27,28,29]. This is especially relevant for natural products because their carrier must first solve the physicochemical limitation of the payload before a targeting ligand can provide additional benefit. For example, a folate- or peptide-functionalised carrier for berberine, nitidine chloride or another hydrophobic alkaloid should be benchmarked against the same non-targeted formulation to determine whether the ligand adds a measurable therapeutic advantage. Such head-to-head comparisons remain an important gap.

### 2.3. Nanosystems as Carriers of Anticancer Agents

**Figure 6:**
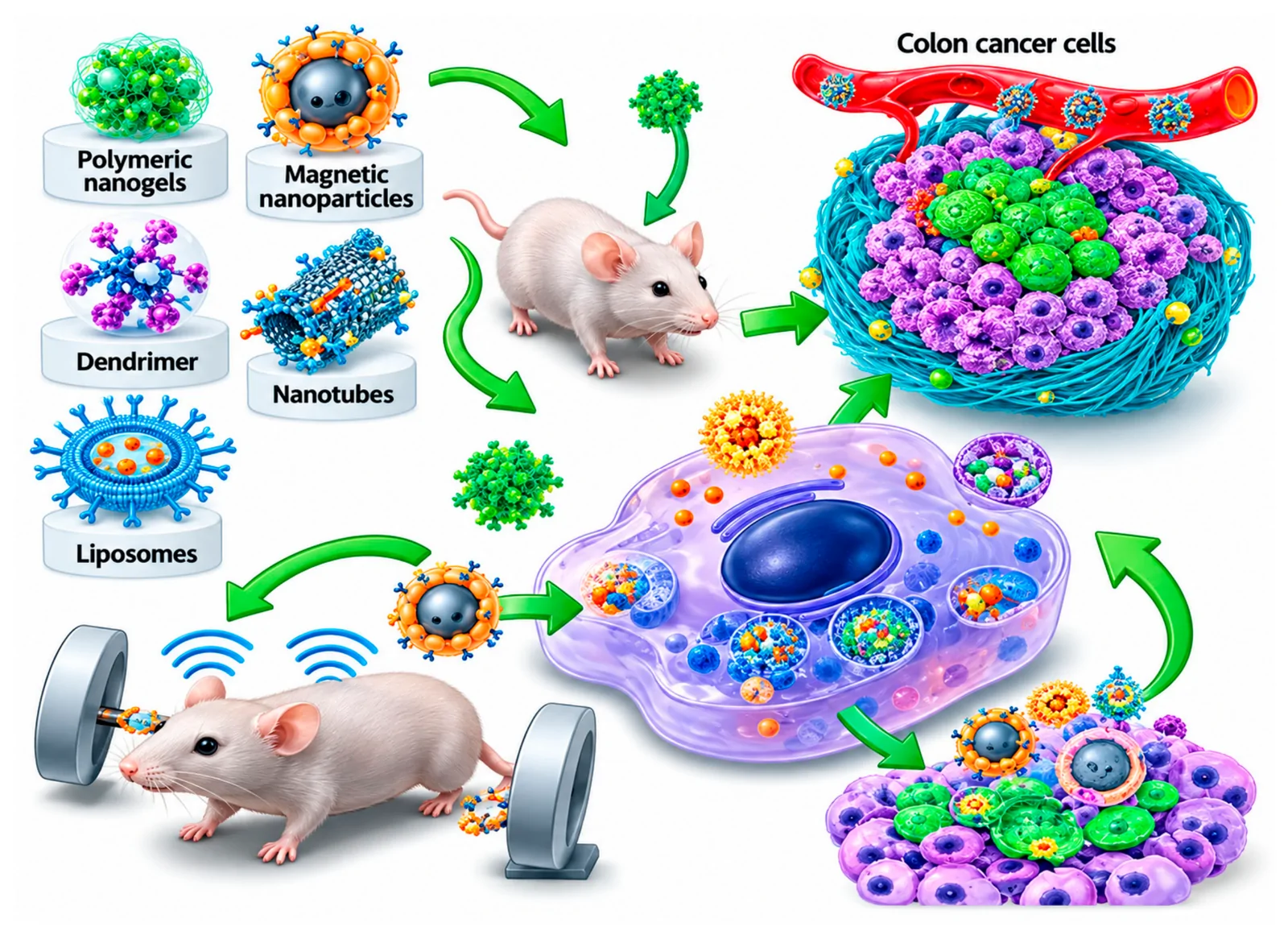
Possible nanodrug delivery systems and their respective approaches in cancer therapeutics for in vitro and in vivo application.

Nanocarriers can improve the performance of anticancer agents by modifying solubility, protecting labile molecules from premature degradation, prolonging circulation, and controlling release. Their behaviour is determined by carrier composition, particle size and morphology, surface chemistry, drug loading, and the biological environment [13,14,18]. Recent literature emphasises that nanocarrier evaluation should extend beyond cytotoxicity to formulation stability, drug-release kinetics, biodistribution, toxicity, pharmacokinetics, and translational feasibility [14,35,36,37,38,39,53]. Figure 6 illustrates the existing nanodrug delivery platforms that can be manipulated to achieve the desired therapeutic outcome(s).

No single nanocarrier platform is optimal for all CRC applications. Lipid systems offer biocompatibility and a relatively mature translational history but may suffer from leakage, instability, and manufacturing constraints; polymeric systems provide structural flexibility and controlled release but require careful control of polymer properties and degradation products; dendrimers provide highly tunable and multifunctional surfaces but can present toxicity, clearance, and synthesis challenges; inorganic systems may provide imaging or externally triggered functions but raise additional questions regarding persistence and long-term safety [13,14,35,37,46,47,55,56].

This distinction is important when interpreting preclinical studies. Improved cytotoxicity in vitro does not necessarily indicate superior clinical performance, because tumour penetration, pharmacokinetics, immune interactions, manufacturing reproducibility, and regulatory requirements may determine whether a formulation can progress beyond laboratory models. Recent nanocarrier studies reinforce the need to assess these factors together rather than treating enhanced cellular uptake or tumour accumulation as sufficient evidence of clinical value [35,36,37,38,39].

The principal platform-specific evidence and applications in CRC are discussed in Section 3, while Section 4 summarises selected preclinical systems, and Section 6 considers additional delivery architectures. Based on the period from 2010 to 2026, the research seems to suggest that future CRC nanomedicine should move from platform-centred development toward disease- and payload-centred design. A rational programme for a natural product should begin with physicochemical and ADME (Absorption, Distribution, Metabolism and Elimination) profiling, then selecting the simplest carrier capable of correcting the identified limitation, followed by comparison with the free compound and an established formulation. More complex features like ligands, stimuli-responsive release, imaging, or co-delivery should be added only when they produce a demonstrable gain in tumour exposure, therapeutic index, or resistance control [14,16,35,36,37,38,39,53]. This approach could reduce unnecessary formulation complexity and improve the probability of translation.

## 3. Nanotechnology in the Treatment of Colorectal Cancer

Nanotechnology-based treatment strategies for CRC increasingly combine carrier engineering with biological targeting, controlled release, imaging, and combination therapy. Rather than treating passive and active targeting as interchangeable concepts, current development increasingly considers how targeting, payload properties, pharmacokinetics, release behaviour, and formulation manufacturability interact. Recent nanomedicine literature also emphasises safety, reproducibility, scalable manufacture, and regulatory compatibility as determinants of clinical translation [14,16,22,35,36,37,38,39,55,57,58,59,60,61,62].

RES-mediated clearance occurs when nanoparticles are recognised and engulfed by macrophages, leading to their removal from the systemic circulation [51]. To mitigate this effect, various surface-modification strategies have been developed. One of the most effective approaches is coating nanoparticles with polyethene glycol (PEG), which significantly reduces opsonisation and prolongs circulation time [52]. Additionally, the physicochemical characteristics of nanosystems, particularly their size and shape, are key determinants of successful drug delivery to colorectal cancer (CRC) sites. For instance, nanoparticles larger than 500 nm are more likely to be rapidly eliminated from circulation. Among the most effective targeting strategies in CRC therapy is the use of monoclonal antibodies, which enable precise, site-specific drug delivery. Representative examples of such systems are summarised in Table 1 and Table 2.

Early CRC nanomedicine studies largely established proof-of-concept for liposomes, polymeric particles, chitosan carriers, and ligand-mediated targeting. During the later 2010s, pH-responsive systems, peptide/antibody targeting, co-delivery, and multifunctional particles became increasingly prominent [63,64,65,66,67,68,69,70,71,72,73,74,75,76,77,78]. From 2021 onward, the literature increasingly emphasises intelligent targeting, biomimetic systems, natural-product payloads, responsive release, theranostic functions, and translation-related quality attributes [11,14,16,22,23,24,25,26,27,28,29,30,31,32,33,34,35,36,37,38,39,53]. The critical lesson from this progression is that technological novelty has outpaced comparative evidence: many studies have demonstrated that a new formulation works, but fewer have established whether it is better than a simpler carrier or that the benefit survives in vivo pharmacokinetic, toxicity, and manufacturing assessments.

**Table 2:** Selected nanoplatform configurations reported in preclinical colorectal cancer research [14,22,35,36,37,38,39,55,62,79,80,81,82,83,84].

Formulation	Ligand	Target
Nanoparticle	Antibody	Carcinoembryonic antigen (CEA)
PEG- and dextran-coated supermagnetic iron oxide nanoparticles	Single-chain Fv antibody fragment (scFv)	Carcinoembryonic antigen (CEA)
Hydroxypropyl-copolymer-doxorubicin conjugates	Peptide GE11	HT29 colorectal cancer
Gold and iron oxide hybrid nanoparticles	Single-chain Fv antibody fragment (scFv)	A33 Antigen
Polymer capsules	Humanized A33 monoclonal antibody	Fas receptor
Mesoporous silica nanoparticles	Coated with poly-(L-lysine) and hyaluronic acid	HCT-116 cancer cells
Chitosan nanoparticles containing 5-aminolevulinic acid	Folic acid	HT29 colorectal cancer cell lines overexpressing folate receptors.
Polymeric dendrimer (PAMAM and PEG)	AS1411-Aptamerand Camptothecin	Human colon adenocarcinoma (in vitro/in vivo)

The platform-specific examples below are interpreted in relation to loading capacity, release control, targeting flexibility, biocompatibility, clearance, manufacturability, and translational readiness rather than as isolated demonstrations of efficacy.

### 3.1. NDDS Used for the Treatment of CRC

#### 3.1.1. Nanoliposomes

Gaballa et al. [85] reported that nanoliposomes (NLs) were the first NPs used in medicine in 1961. At the time, NLs were the first drug delivery system (DDS) approved for clinical applications. Nanoliposomes are widely used delivery systems (DSs) for small molecules, nucleic acids (short- and long-base-pair sequences), proteins, and peptides. In terms of morphology, NLs are small, spherical, synthetic particles composed of a phospholipid bilayer and an aqueous core, and are generally non-toxic [86,87]. Because they have a phospholipid bilayer, are 100-200 nm in size, and can incorporate various compounds, NLs are among the most effective DDSs for targeted therapy. In addition, they have favourable properties, such as slow release of the active drug within the tumour, the ability to reduce side effects, and site-specific delivery [88].

**Figure 7:**
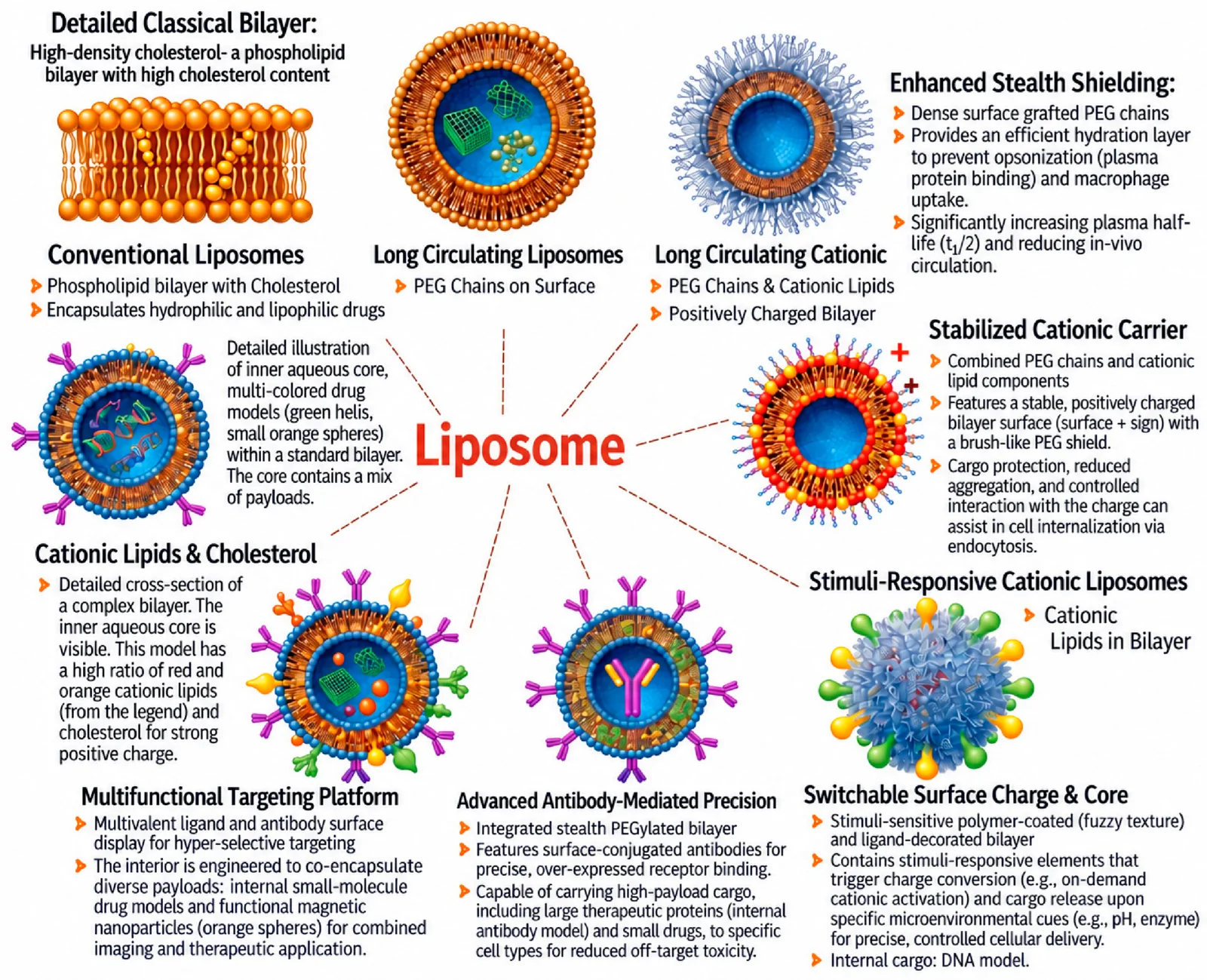
Surface modification of lipid-based nanoparticles for the active targeting of CRC therapy. The cross is actually a plus sign for positively charged.

An NL system is developed based on three intended outcomes: long circulation, active targeting, and stimuli-responsive NLs, as illustrated in Figure 7. For long-circulating LPs, their surfaces are strongly affected by opsonization, the RES takes up the opsonised LPs, and rapidly clears them from the circulation [87]. Modifying LPs by attaching PEG tends to mask NPs from blood plasma opsonins, allowing them to evade RES recognition and prolong their half-life. On the other hand, active targeting would involve LPs targeting glycosidic residues, receptors, and peptides. Another approach would be to design LPs that are light-, temperature-, magnetic-, or pH-responsive. One of the first LP formulations approved by the Food and Drug Administration (FDA) contained the anticancer drugs doxorubicin (Doxil^®^) and daunorubicin (DaunoXome^®^) in the 1990s. The drug Doxil^®^ has a size of 100 nm and exhibits low toxicity towards the heart and gastrointestinal tract. In 2012, the LP drug Marqibo^®^ [16] was approved by the FDA for cancer therapy. Marqibo is approximately 100 nm in size and targets the cell cycle. Efforts have been made to address drug resistance when using LP-based drugs such as doxorubicin. When administering high dosages of an LP-based DDS for doxorubicin, such as the L33 (an aptamer-based DDS), the LP-based DDS can deliver the drug to and into target cells [21]. Thermodox^®^, a thermo-responsive form of doxorubicin, is another type of LP-based DDS [89]. This DDS is currently in a Phase II trial that seeks to treat combined colon and liver metastases using radiofrequency ablation (RFA). Thermodox^®^ has been reported to deliver 25 times the amount of doxorubicin into cancer cells compared with intravenous (IV) doxorubicin and 5-fold more than standard LP-based formulations of the drug in vivo [90].

Liposomes remain attractive for compounds such as curcumin, apigenin, and other poorly soluble natural molecules because their lipid bilayers can accommodate hydrophobic payloads while permitting surface modification [2,21,89]. However, their long history also exposes persistent problems drug leakage, oxidation, storage stability, and manufacturing consistency. The innovation opportunity is therefore not simply another liposome, but formulations in which the release profile and targeting mechanism are matched to the biological limitation of the selected natural product and validated against free drug and non-targeted liposome controls.

#### 3.1.2. Dendrimers

**Figure 8:**
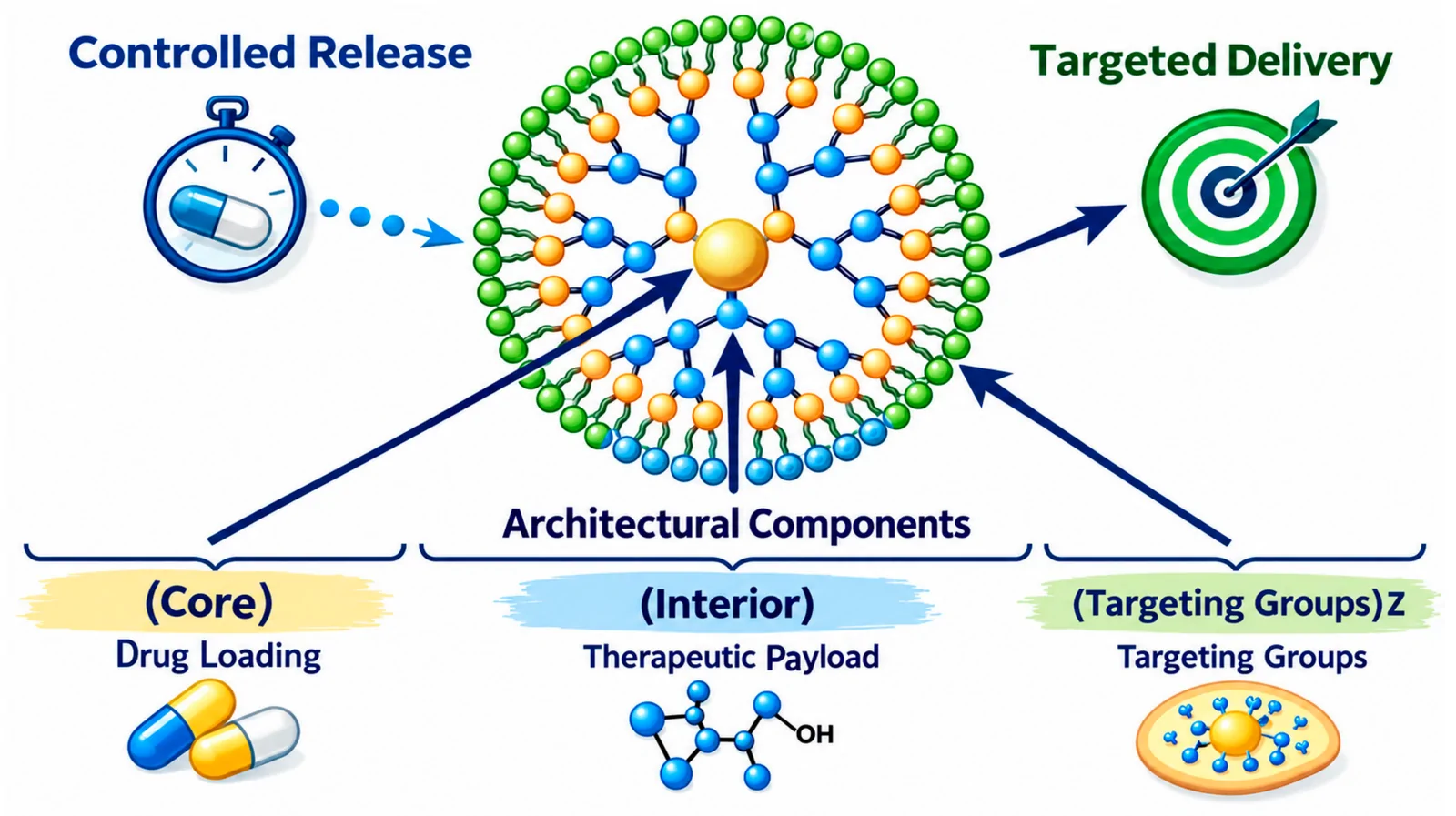
Architectural design of dendrimers in cancer therapeutics.

Dendrimers were first described by Fritz Vogtle and colleagues in 1978 [91]. They are highly branched, tree-like polymeric nanostructures. Owing to their nanoscale dimensions, good water solubility, biocompatibility, and uniform (monodisperse) nature, dendrimers have attracted considerable attention for drug delivery applications. These nanostructures typically comprise three key components: a central core, repeatedly branching dendritic units, and terminal surface functional groups, which can be either hydrophilic or hydrophobic, as illustrated in Figure 8. Recent work on dendrimer-based nanocarriers illustrates continued development towards multifunctional systems with improved intracellular delivery and controlled therapeutic responses [35,37]. These studies also reinforce that added structural complexity should be justified by demonstrable therapeutic benefit and acceptable safety.

**Figure 9:**
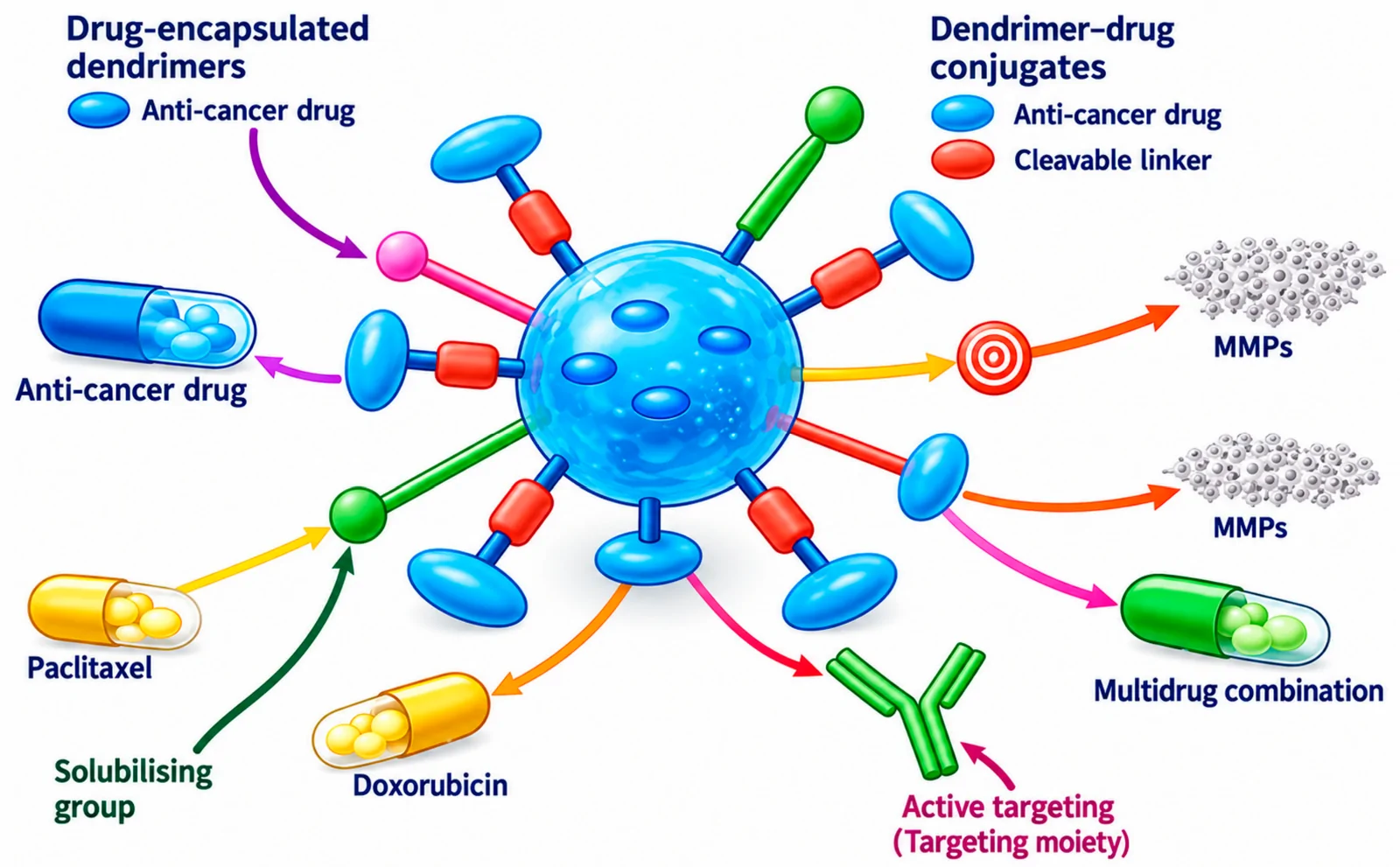
Surface modification of dendrimers for the active targeting of CRC therapy.

These surface-active groups are integral to determining the dendrimer’s physicochemical attributes. Dendrimers’ branched nature affords them their versatility and enables their use in drug delivery [92,93]. In addition, they exhibit higher encapsulation efficiencies due to the surface-active groups facing outward. Dendrimers are synthesized using two approaches: divergent and convergent methods [94]. Dendrimers are available as dendritic hydrocarbons, silicone-based dendrimers, polylysine dendrimers, poly(propylene-imine) (PPI), POPAM (a combination of polyamidoamine and polypropylene-imine), and polyamidoamine (PAMAM), phosphorus-based dendrimers, porphyrin-based polymers, dendric hydrocarbons, Newkome dendrimers, and carbon/oxygen-based dendrimers [95]. Newkome’s arboreal system and Tomalia’s PAMAM are the most studied dendrimers, attracting significant interest [94]. In Figure 9, the various surface modification areas of interest are shown together with their respective anticancer molecules.

Unfortunately, traditional dendrimers are rapidly cleared by the RES, thereby reducing their uptake by CRC cells. Overcoming these drawbacks is essential. Modifications can be achieved by hybridization with other NSs, copolymerization with a linear polymer such as PEG, or chemical modification. For active targeting of cancer cells, the dendrimer’s surface can be enhanced by conjugating peptides, antibodies, proteins, aptamers, and carbohydrates. The surface can also be modified with compounds responsive to pH, light, UV, electromagnetic fields, enzymes, ultrasound, and temperature [96,97]. Nanotechnology used for the delivery of genetic material for cancer treatment is highly significant and very useful. For example, PAMAM, due to its cationic nature, can be used to deliver genetic material for DNA repair therapy. Haensler and Szoka used PAMAM to deliver nucleic acids [98]. Recent dendrimer studies further demonstrate the field’s movement towards multifunctional and stimuli-responsive architectures, although these approaches remain largely preclinical and require a careful assessment of safety, biodistribution, and manufacturability [35,37].

Dendrimers offer a higher degree of structural control than many conventional carriers, making them attractive for multivalent targeting and co-delivery. Nevertheless, the 2010–2026 literature also shows why complexity must be justified: surface charge, generation, branching architecture, and ligand density can alter cellular toxicity, biodistribution, and clearance [35,37,56,93,94,95,96,98]. For plant-derived molecules such as berberine or nitidine chloride, the key unanswered question is whether dendrimer-mediated gains in intracellular delivery are large enough to offset synthesis complexity and safety concerns. Direct comparisons with liposomes and PLGA systems are therefore a priority for future CRC studies.

#### 3.1.3. Superparamagnetic Iron Oxide Nanoparticles (SPIONs)

**Figure 10:**
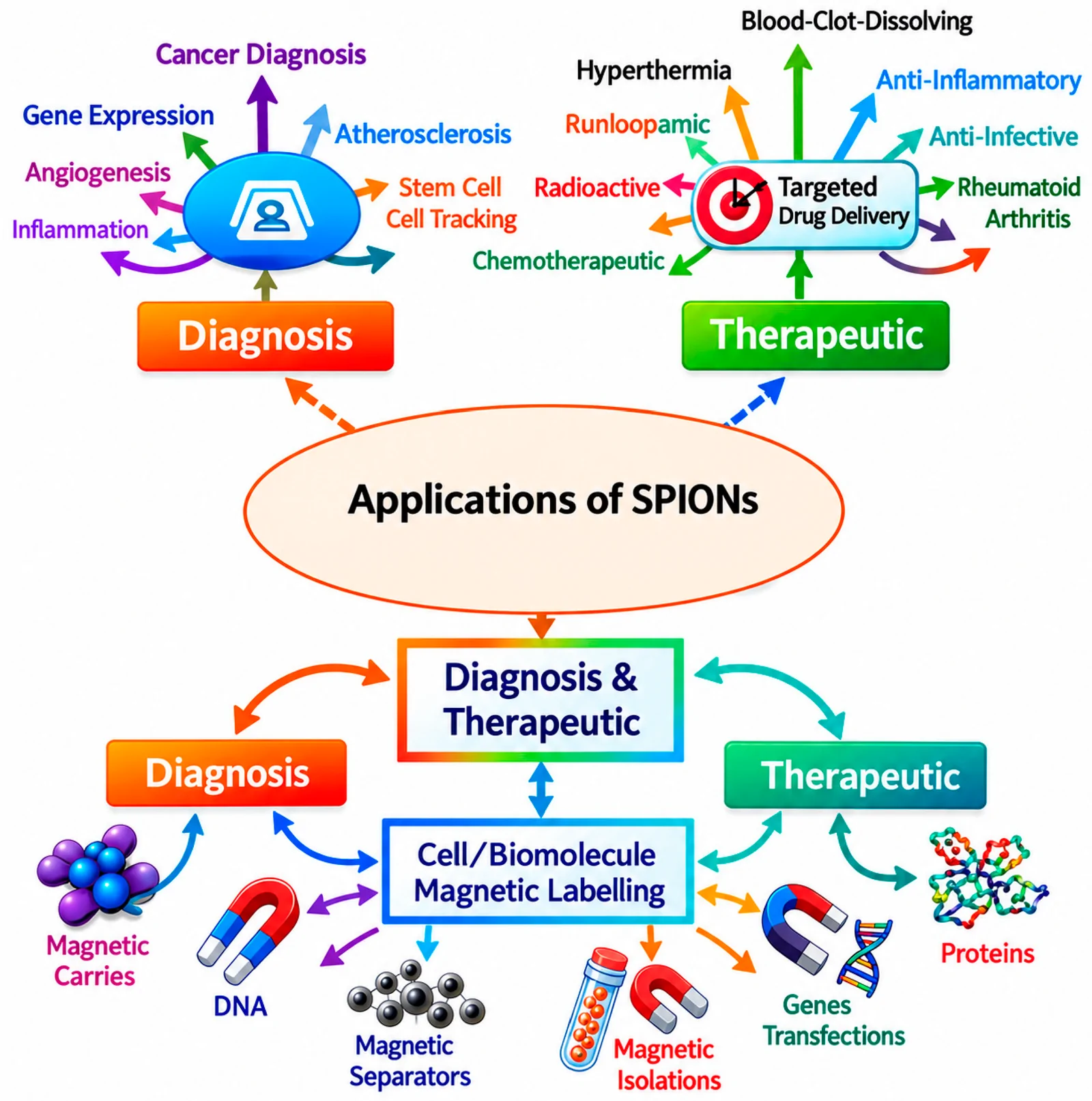
Flo chart of the application of SPIONs in nanomedicine.

SPIONs are a type of nanocarrier (NS) that has attracted considerable attention for drug delivery since 1980 [99]. Over the years, they have been engineered with biocompatible polymer coatings and surface modifications of their core. Their therapeutic and diagnostic uses are outlined in the flowchart depicted in Figure 10. Key features of this delivery platform include magnetic properties that require an external magnetic field for guidance. Additionally, drugs and medical radioisotopes can be loaded into SPIONs. These NSs have revolutionized nanomedicine and nuclear medicine over the past few decades. Drug-loaded and radiolabeled SPIONs have greatly enhanced the detection and treatment of CRC and other cancers. Their main benefits include longer circulation time, improved pharmacokinetics, high drug-loading capacity, reduced side effects, and targeted delivery to cancer tumors [100]. The advantages of SPIONs include prolonged circulation, targeted delivery, and minimized side effects.

SPIONs doped with gold have been developed for targeted photothermal therapy to destroy CRC cells [101]. These gold-doped SPIONs were also functionalized with an antibody to enable active targeting of the transmembrane glycoprotein (A33) antigen, which is overexpressed in CRC cells. Studies evaluating the anticancer potential of the gold-doped SPIONs revealed that the accumulation of the gold-SPIONs inside CRC cells was 5-fold higher in cells that expressed the A33 antigen than in those that did not. The study further revealed that when laser radiation was applied to the gold-SPIONs, 53% of cells expressing the A33 antigen were destroyed. In contrast, only 5% of those that did not express the antigen were destroyed. These results demonstrate SPIONs’ ability to differentiate between normal and healthy cells. In addition, a small percentage of normal cells were destroyed.

SPIONs that are antibody or peptide-based act as active transporters, targeting drugs to cancerous organs or tissues [102]. In contrast, passive SPIONs rely primarily on polymer type and particle size to enable NS accumulation at the delivery site. Hydrophilic SPIONs, such as those coated with polyethene glycol or dextran, can avoid uptake and elimination by the RES, thereby increasing their biological half-life. Another design approach uses SPIONs smaller than 30 nm, as they can extravasate from the vascular space into the interstitial space, where macrophages or monocytes may take them up and transport them to the lymph nodes via the lymphatic vessels [103].

Metallic nanoparticles provide functions that conventional organic carriers cannot readily reproduce, including plasmonic imaging and photothermal effects [55,62,104]. Yet the same properties that create multimodal functionality can introduce persistence, surface-dependent toxicity, and clearance questions. The plant-extract-mediated synthesis literature should also be interpreted cautiously: biological activity of a green-synthesised nanoparticle does not establish which phytochemicals remain on the surface, how reproducible the formulation is, or whether the nanoparticle or residual extract drives the observed effect. Standardised chemical characterisation and matched controls are therefore essential before such systems can be considered credible delivery platforms [105,106]. SPIONs are distinctive because magnetic guidance can provide a controllable physical input that passive EPR cannot. However, the requirement for an appropriate external field, magnetic penetration, particle retention, and long-term iron handling makes translation more demanding than for simpler carriers [100,102,107]. Their strongest future role may therefore be in combination strategies, for example, when a natural-product payload also benefits from image guidance or externally triggered release, rather than as a default carrier for every plant compound.

#### 3.1.4. Metallic Nanoparticles

**Figure 11:**
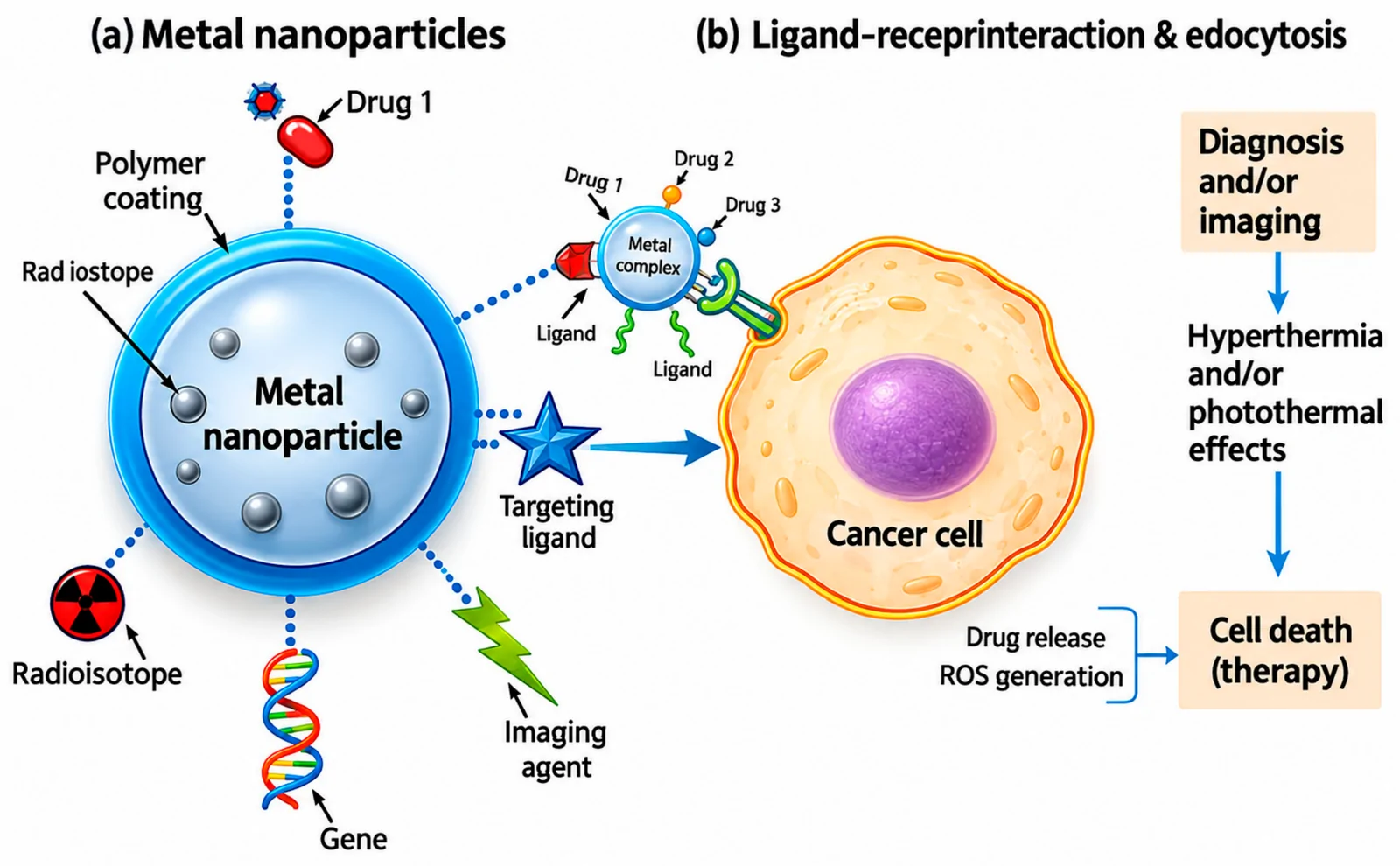
Nanoparticle surface modification and targeting approach used in nanomedicine.

Metal oxide and metallic nanoparticles (MNPs), which can be tuned for biocompatibility, shape, size, and surface derivatization, play a crucial role in drug delivery [104,108]. Gold nanoparticles (AgNPs) have shown significant potential as anticancer agents for treating various cancers. They have been used as drug delivery systems in colon cancer therapy, where combining AgNPs with drugs enhances anticancer effectiveness. These systems act as delivery vehicles and are non-toxic to healthy cells. Yan et al. [107] synthesized AgNPs using pollen extract from *P. dactylifera* and leaf extracts from I. hirsute to target different cancers. In CRC, both extracts demonstrated dose-dependent cytotoxicity. Coating AgNPs with anticancer drugs is another promising strategy, as it can confer sustained-release properties. Dey et al. [109] created AgNPs coated with a camptothecin-based prodrug, which showed pH-controlled, sustained drug release. Such MNP-based drug delivery systems (DDS) can inhibit and destroy cancer cells, potentially preventing tumor metastasis. Developing MNPs with favorable physicochemical properties and anticancer drug coatings may also help overcome tumor drug resistance. Figure 11 illustrates the application of metallic nanoparticles in cancer therapy.

Metallic systems should be reserved for cases where their optical, photothermal, or imaging functions provide a specific therapeutic advantage. For plant-derived payloads, claims of synergistic cytotoxicity require controls that separate nanoparticle effects, ligand effects, and natural-product effects. Long-term accumulation and clearance remain central translational barriers [22,55,62].

#### 3.1.5. Mesoporous Silica Nanomaterials

Mesoporous silica nanomaterials (MSNs) are another class of NS used for treatment. MSNs have favorable surface properties, including stability, a large surface area, a narrow size distribution, and uniform porosity [110]. The ease of surface modification facilitates the development of multipurpose DDSs using MSNs. This research has led to the loading of therapeutic agents onto MSNs for delivery to cancer cells or tissues [111]. The application of many antineoplastic agents for effective cancer therapeutics is often limited by their stability, bioavailability, and solubility. Shahbaz et al. [112] designed a novel NS for cancer tissue targeting to overcome these drawbacks. Their study modified the surface of MSNs by introducing curcumin, while another MSN modification contained hyaluronan. These modifications were designed to deliver polyethyleneimine-folic acid. The MSNs were tested on mouse xenograft models. The folic acid-modified MSNs showed significantly enhanced cellular uptake compared with that of the non-targeted MSNs. The folic acid-modified MSNs also showed improved biocompatibility and low toxicity. These MSNs further inhibited tumor cells compared with the curcumin formulation. A study by Xu et al. [113] developed a microporous silica DDS to treat multidrug-resistant cancer cells with elevated B-cell lymphoma 2 (Bcl-2) levels. Their system exhibited a 40% peptide loading efficiency for the Bcl-2-converting peptide (derived from orphan nuclear receptor Nurr77). Upon introduction to the cancer cells, apoptosis was induced.

MSNs can be engineered to respond to pH changes. This enables the release of drugs or bioactive agents trapped within the pores at target sites in response to environmental pH shifts, thereby regulating the release of active pharmaceutical ingredients (APIs). Such stimuli-responsive systems have been used in the fight against CRC. Li et al. developed s hollow mesoporous silica NS to deliver DOX to HeLa (Henrietta Lacks) cancer cell lines. The study was conducted under acidic pH conditions, and the NS exhibited significant biocompatibility. They further developed hyaluronic acid end-capped MSNs loaded with DOX to target the CD_44_ antigen. The system was pH-responsive and showed improved cellular uptake via CD_44_ receptor-mediated endocytosis in HeLa cells. The findings further revealed that high DOX accumulation occurred in cells overexpressing the CD44 receptor, and not in normal cells [91]. Therefore, these findings indicate that stimulus-mediated drug delivery using MSNs can be a promising active-targeting tool for cancer treatment. A more recent study by Ansari et al. [114] developed a core-shell MSN DDS. The system consisted of a magnetic core and an MSN shell to deliver epirubicin. The study reported a 27% increase in epirubicin uptake by cancer cells treated with magnetic core–shell-MSNs DDS upon exposure to an external magnetic field compared with epirubicin alone.

MSNs are particularly interesting for natural products because their pore architecture can separate loading from surface targeting and permit controlled release [47,110,111,112,113,114,115,116]. However, the literature contains relatively few direct comparisons showing that the higher loading capacity or responsive release of an MSN produces a clinically meaningful advantage over liposomes or polymeric particles. For plant-derived compounds, future studies should quantify loading stability in gastrointestinal and plasma conditions, release of the pharmacologically active free compound, silica clearance, and tumour exposure. This is an area where formulation engineering and pharmacology need to be investigated together rather than sequentially.

**Figure 12:**
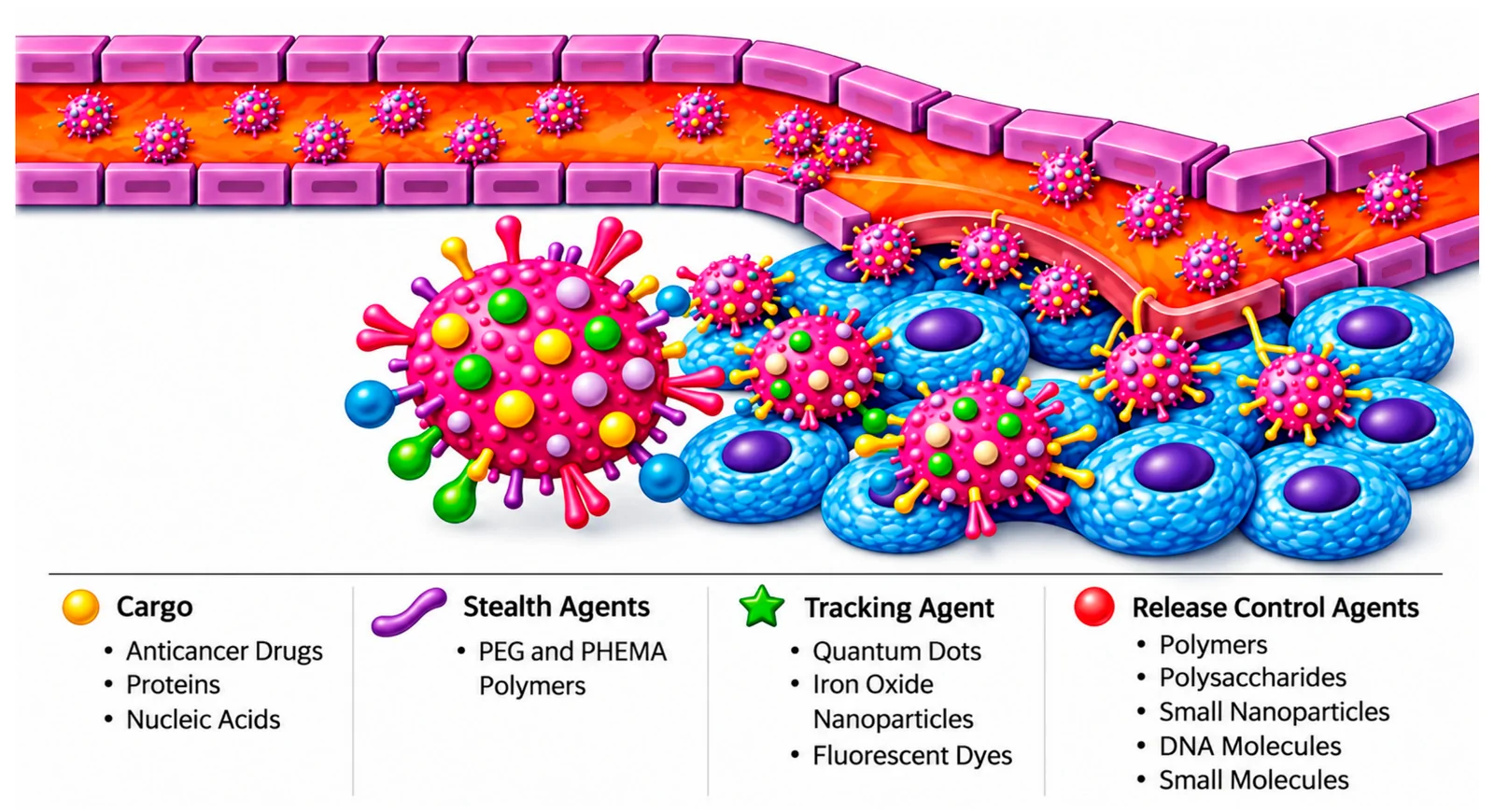
Different methods of loading mesoporous hydrogels.

Versatile MSNs can be combined with various NSs to enhance their overall anticancer properties and reduce cytotoxicity towards normal cells, as shown in Figure 12. These platforms can be designed to be selective and responsive to environmental stimuli, thereby regulating the sustained release of the anticancer drug. MSNs are very promising NSs that in the future could provide an alternative to conventional methods used in cancer therapeutics due to their significant properties. In addition, more studies are required to examine the pharmacodynamic and pharmacokinetic properties of the NS. These findings would help in understanding whether such systems have immense clinical potential [115].

#### 3.1.6. Quantum Dots

**Figure 13:**
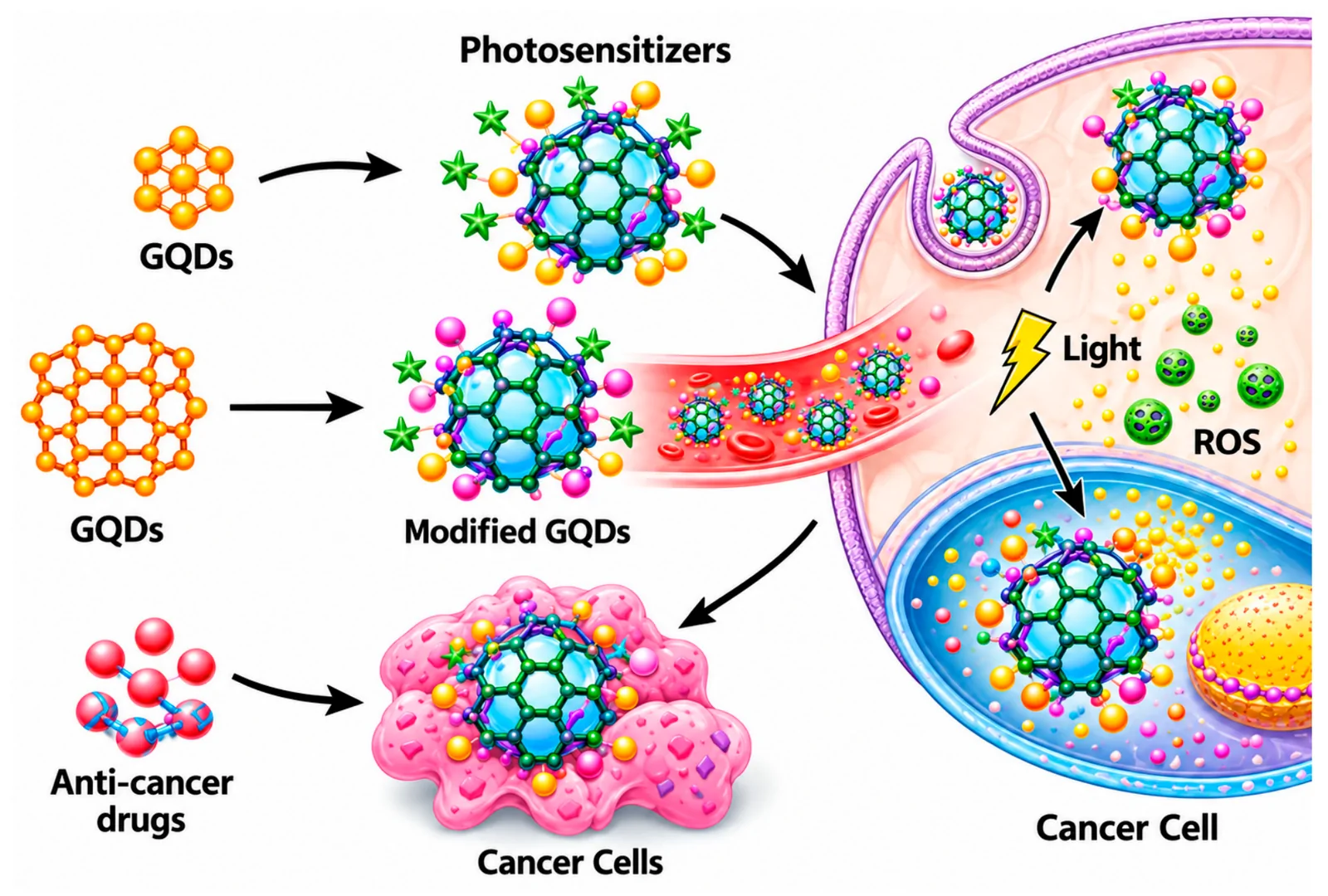
Modification approaches used in QD development for anti-cancer therapy.

Quantum dots (QDs) are nanoscale semiconductor materials known for their fluorescent properties. Typically composed of elements from groups II and VI of the periodic table, these nanostructures exhibit unique photophysical behaviour strongly influenced by their size, which typically ranges from 2 to 10 nm [117]. QDs exhibit several advantageous characteristics, including strong photoluminescence, good biocompatibility, stability in physiological environments, and a high drug-loading capacity, making them attractive candidates for nanomedicine applications. Figure 13 illustrates the mechanism of action of QDs in cancer therapy. As a result, they have been extensively explored as drug delivery systems (DDS) and for biosensing and imaging technologies. Noorizadeh [118] designed a multifunctional QD-based platform for combining cancer diagnosis and therapy. In their study, graphene quantum dots were functionalised and conjugated to polyethene glycol (PEG), a porphyrin derivative, and an aptamer, resulting in a composite system with enhanced fluorescence. Porphyrins and their derivatives are widely recognised as effective photosensitisers due to their ability to generate singlet oxygen and their relatively low toxicity [119].

Upon light activation at specific wavelengths, these photosensitizers transfer energy to molecular oxygen, producing reactive oxygen species (ROS), such as singlet oxygen, which can induce tumour cell destruction through photodynamic effects. Despite these promising features, unmodified QDs often face challenges, including limited stability under physiological conditions, poor cellular internalisation, and hydrophobicity, which limit their therapeutic use. To address these limitations, functionalisation strategies have been employed, including the integration of graphene and subsequent conjugation with porphyrin molecules. These modifications improve biocompatibility, enhance stability in biological environments, and reduce toxicity. Moreover, such engineered systems can distinguish between malignant and healthy cells. Graphene quantum dots (GQDs) offer a large surface area that facilitates the loading and delivery of biomolecules such as nucleic acids, supporting both diagnostic and therapeutic applications. Studies have also reported membrane disruption and apoptosis induction in cancer cells treated with GQD-PEG-porphyrin systems [120,121].

Further functionalisation of GQDs with targeting ligands has been shown to enhance their cellular uptake. For example, conjugation with folate improves receptor-mediated endocytosis in cancer cells compared to non-targeted GQDs. Barati et al. [122] developed antibody-conjugated GQDs by linking a single-chain variable fragment (scFv) to the nanoparticle surface (GQDs-scFvB10), which demonstrated strong affinity for the epidermal growth factor receptor (EGFR). These targeted systems also exhibited high drug-loading efficiency, with reports indicating up to 50% loading of cisplatin (CsPt). Drug release from these nanocarriers was pH-sensitive, with slower release at physiological (neutral) pH and accelerated release in acidic environments. Specifically, approximately 55% of cisplatin was released at pH 5.5 within six hours, compared to about 20% at pH 7.4 over the same period. This pH-responsive behaviour is attributed to the protonation of functional groups on the GQDs, which reduces electrostatic interactions and facilitates drug release. Importantly, cisplatin-loaded, EGFR-targeted GQDs demonstrated enhanced cytotoxicity towards cancer cells, such as MDA-MB-231 cells, compared to the free drug, due to increased uptake mediated by receptor targeting. In contrast, reduced uptake and toxicity were observed in cells with low or saturated EGFR expression, further highlighting the specificity of these nanocarrier systems for tumour cells that overexpress this receptor.

QDs are best viewed as specialised theranostic rather than first-line delivery platforms in this review. Their principal advantage is the integration of imaging with delivery or photodynamic functions, whereas concerns about composition, physiological stability, and long-term safety constrain routine therapeutic use [117,118,119,120,121,122]. A meaningful innovation pathway would be to use QD-based systems only where real-time localisation or externally activated therapy provides information or therapeutic control that simpler natural-product carriers cannot provide.

#### 3.1.7. Carbon Nanotubes

**Figure 14:**
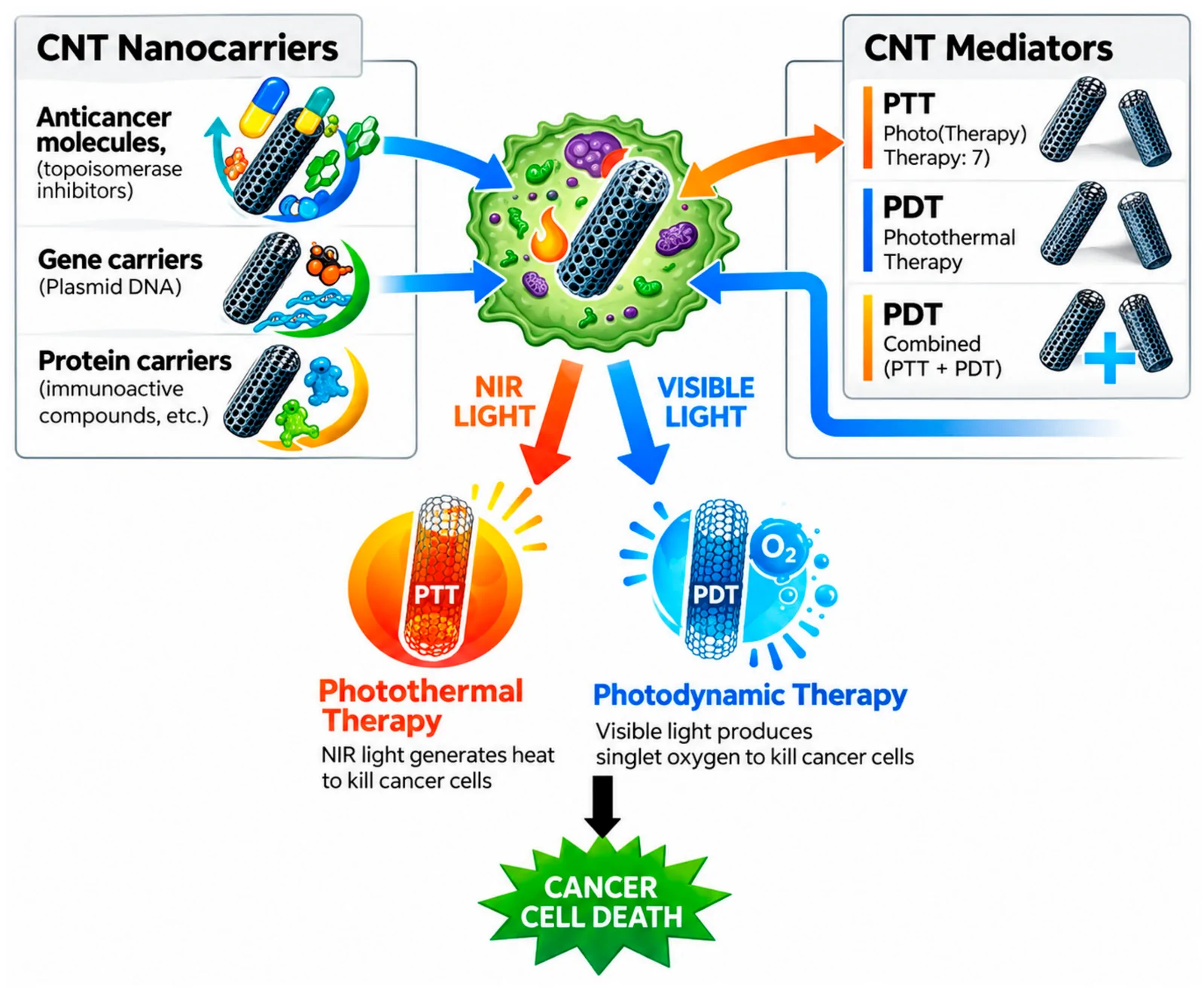
CNT therapeutic approaches used in cancer therapy.

Carbon nanotubes (CNTs) are nanoscale, cylindrical carbon structures composed of interconnected benzene rings. They have been widely explored for drug, peptide, and vaccine delivery, as well as for the diagnosis and treatment of various cancers. Despite poor solubility in most solvents and potential toxicity concerns, CNTs offer several advantages, including a large surface area, high drug-loading capacity, and surfaces that can be readily modified and applied in cancer therapy, as seen in Figure 14 [123]. In oncological research, both graphene-based materials and CNTs have attracted considerable attention. Limitations associated with CNTs, particularly their insolubility, can be addressed through chemical modification, which enhances their dispersibility in aqueous environments. Furthermore, CNTs can be functionalised with a range of biomolecules, including peptides, proteins, nucleic acids, and therapeutic agents, thereby improving their applicability in biomedical contexts [124]. The application of CNTs also supports the targeted and site-specific delivery of anticancer drugs to tumour cells. This is especially important for overcoming multidrug resistance, a major challenge in cancer treatment driven by multiple cellular mechanisms of resistance [125]. Anticancer agents such as methotrexate have been functionalized with CNTs via covalent linkage to the fluorescent agent fluorescein isothiocyanate (FITC). In vivo investigations of these NSs, which contained anticancer drugs bound to them, showed significant accumulation and internalization of the drug in tumor cells compared with free drugs alone. The introduction of multi-walled carbon nanotubes (MWCNTs) has shown significant efficacy in multidrug-resistant tumor cells [126]. MWCNTs can co-deliver drugs, enable site-specific targeting, and achieve high uptake by cancer cells, even those that are resistant. Wang et al. produced MWCNTs that exhibited an excellent circulation half-life, a high drug-loading ratio, and active targeting capabilities. It was further reported that pH-responsive CNT platforms produce heat after light absorption when irradiated with near-infrared light (NIR). Since DOX has significant toxicity towards cancer cells and further exhibits a multimodal nanomedicine effect, this combination with MWCNTs can help achieve effective, efficient cancer cell treatment.

Nazir et al. [127] designed a single-walled carbon nanotube (SWCNT)-based drug delivery system (DDS) by conjugating human serum albumin (HSA) nanoparticles to load the anticancer drug paclitaxel (PTX). The developed SWCNT DDS demonstrated high efficiency in intracellular drug delivery, with about 80% cellular uptake in cancer cells, as confirmed by fluorescence-labelled carriers. Their findings indicate that this needle-shaped nanostructure can transport the anticancer agent through the cell membrane despite its complex macrostructure. The drug-loading capacity was primarily driven by the strong binding affinity between HSA and PTX. The HSA-PTX SWCNTs showed significantly greater tumor inhibition than the HSA SWCNTs without PTX, and substantial drug accumulation within tumor cells was also observed.

CNTs illustrate the tension between high loading capacity and translational safety. Their large surface area and functionalisation potential can improve intracellular delivery, but insolubility, persistence, and toxicity concerns make them less immediately attractive than biodegradable lipid, polymeric, or chitosan systems for natural products [123,124,125,126,127]. For CRC, the strongest research case is therefore likely to involve highly controlled, surface-engineered CNTs for specialised applications such as combination or externally activated therapy, accompanied by rigorous long-term biodistribution and clearance studies.

#### 3.1.8. Chitosan Nanoparticles

Nanoparticles from chitosan (CNPs) have attracted increasing attention for therapeutic use, including the delivery of proteins, genes, and anticancer agents. These NSs can be administered via the oral, nasal, intravenous, and ocular routes [128]. By 2015, Shashank and Tummala produced chitosan 5-FU NPs to reduce cytotoxicity in somatic cells and to specifically target the anticancer agent within the colon. Their study revealed that their CNPs enhanced the internalization of 5-FU within the tumor. They also observed sustained 5-FU release over 24 h. This reduced the toxicity of anticancer drugs to normal cells, as a large amount of 5-FU was localized in the CRC cells. These findings indicate that the patient benefits, as the dosage frequency and 5-FU administration are decreased [129]. In another study, Tan et al. [130] produced chitosan CNPs loaded with 5-FU to achieve enhanced localization and targeting efficiency of 5-FU to cancer cells. The in vitro results of their release studies revealed controlled, sustained release of the active drug from the CNPs, with release rates of 29-60% under varied pH conditions over an incubation period of 48 h. In conclusion, the authors suggested that the drug to be encapsulated in CNPs should be evaluated as a pH-responsive smart DDS for cancer treatment [131,132,133].

Chitosan occupies an important translational niche because it combines biodegradability, mucoadhesive properties, and compatibility with oral or colon-targeted delivery. The continuing development of 5-FU-loaded chitosan systems into 2026 suggests that relatively simple biomaterials can remain competitive when the clinical objective is localised gastrointestinal delivery [40,128,129]. This is relevant to natural products: an oral chitosan formulation may be more rational for a poorly absorbed plant compound intended to act locally in the colon than a highly complex intravenously administered nanoplatform. Comparative studies should therefore include route of administration as a core design variable.

#### 3.1.9. Nanoemulsion System and Polymeric Nanoparticles

A nanoemulsion (NE) is a clear, thermostable mixture of oil, water, and surfactant with consistent physical characteristics [134]. NEs have advantageous features, such as safeguarding anticancer drugs from environmental factors that could degrade or destroy them and enabling the dissolution of non-polar compounds. They are known for being highly stable, efficient, and low toxic. Bano et al. [135] studied the anticancer effects of gold nanoparticles (AuNPs) and lycopene (LP) against colon cancer cell lines (HT-29). Their findings indicated that AuNPs and LP within nanoemulsion systems caused apoptosis three times more effectively than gold nanoparticles and lycopene alone [136].

Polymeric nanoparticles (PNPs), typically 10–100 nm in diameter, are nanoscale systems. To avoid detection and removal by the immune system, they are usually coated with non-ionic surfactants, which reduce processes such as opsonisation [137,138]. The most studied synthetic polymers include poly(lactic-co-glycolic acid) (PLGA) and polycaprolactone (PCL), both approved by the Food and Drug Administration (FDA) for biomedical use [139]. 5-Fluorouracil (5-FU) remains a primary chemotherapeutic agent for colorectal cancer (CRC). Despite its effectiveness against cancer cells, it can also harm healthy tissues and shows limited accumulation at tumour sites [2].

Dhara et al. [2] investigated the use of citrus pectin and Eudragit S100, a pH-sensitive polymer, to develop nanoparticle delivery systems to improve 5-FU targeting in CRC cells. Their results showed that pectin-based carriers effectively delivered the drug, increasing its accumulation in cancer cells [2]. Additionally, in vitro and in vivo tests on Eudragit S100-coated nanoparticles revealed stability under acidic conditions (pH < 7) and sustained drug release over time [110]. Citrus pectin also acted as a targeting ligand for galectin-3 receptors, which are overexpressed in CRC cells, thereby supporting site-specific drug delivery [110].

Across the platforms reviewed above, the evidence supports a shift from single-function carriers towards systems that combine controlled release, tumour recognition, imaging or externally triggered therapy, and improved pharmacokinetics. However, platform selection should remain payload- and disease-specific: the advantages of high loading, long circulation, ligand functionalisation, or external responsiveness must be weighed against toxicity, clearance, stability, scale-up, and regulatory complexity. Recent nanomedicine literature reinforces the need to evaluate drug-release behaviour and in vivo performance together, rather than relying solely on cell-based cytotoxicity [14,35,36,37,38,39,55]. For natural products, nanoemulsions and polymeric nanoparticles are especially useful as benchmark platforms [140] because they can address poor aqueous solubility without necessarily requiring the extensive surface engineering of more complex systems. The 2025–2026 literature also supports a quality-by-design direction in which formulation variables, release kinetics, and biological performance are linked systematically rather than optimised independently [2,13,14,53,141]. Future studies should therefore report a clear comparator hierarchy: free compound, simple carrier, targeted carrier, and where justified, multifunctional carrier. This would make it possible to determine whether added nanotechnology produces incremental therapeutic value.

### 3.2. Comparison of Nanocarrier Platforms

Across the platforms reviewed in Section 3, liposomes offer strong biocompatibility, flexible loading of hydrophilic and lipophilic agents, and relatively mature clinical experience, but leakage, storage stability, and manufacturing cost can limit performance. Polymeric nanoparticles and micelles provide greater control over loading and release and are particularly useful for poorly soluble small molecules, although polymer composition, degradation products, and batch-to-batch reproducibility require close control. Dendrimers offer highly defined, multifunctional surfaces and opportunities for ligand attachment and co-delivery, but toxicity, clearance, and synthetic complexity remain important limitations. Metallic and magnetic nanoparticles provide imaging, magnetic guidance, photothermal, and other multimodal functions, but long-term clearance and safety require careful evaluation. Mesoporous silica nanoparticles provide high surface area and tunable pore architecture, whereas biodegradation and persistence remain relevant concerns. Exosome-based systems offer biomimetic interfaces but face major challenges in isolation, loading consistency, standardisation, scalability, and regulatory control. Hybrid lipid–polymer systems combine complementary properties but introduce additional formulation complexity. Overall, platform selection should be based on the physicochemical properties of the payload, desired release profile, biological target, route of administration, safety profile, and translational feasibility rather than on in vitro potency alone [14,22,35,36,37,38,39,46,47,55,56,62,116]. Taken together, the evidence from 2010–2026 does not support declaring a universal “best” nanocarrier for CRC. Lipid systems generally offer the most familiar biocompatibility and formulation logic; polymeric and chitosan systems provide useful control of release and colon-directed administration; dendrimers offer precision at the expense of complexity; metallic, magnetic, and quantum systems add externally controlled or imaging functions; MSNs offer high loading and programmable pores; and biomimetic or hybrid systems seek to combine advantages from several classes. The research gap is therefore comparative rather than purely technological: head-to-head studies using the same natural product, dose, route, and tumour model are needed to establish which platform provides the best therapeutic index.

## 4. Nanosystems Currently in Preclinical Studies for the Treatment of Colon Cancer

A variety of nanoscale formulations are being explored for diagnosing and treating colorectal cancer (CRC). Recent research has tested these systems in in vivo CRC models, with several examples summarised in Table 3. The most common preclinical model involves inducing subcutaneous colon tumours in BALB/c mice using cell lines such as CT26, C26, and HCT116. Fewer studies, however, have developed orthotopic CRC models within the intestinal mucosa, for instance, using Caco-2 cells in C57BL/6 mice [142]. Liposomal and PEGylated polymeric nanoparticles are frequently used, especially in therapy, while inorganic nanostructures are also widely studied for diagnostic and therapeutic purposes.

In many cases, nanosystems are engineered for active targeting. This can be achieved either through magnetic components such as superparamagnetic iron oxide nanoparticles (SPIONs), silver, or gold, guided by external magnetic fields, or through the conjugation of targeting ligands, including folate, cyclic RGD (cRGD) peptides, epidermal growth factor receptor (EGFR)-targeting molecules, and hyaluronic acid. These nanocarriers are commonly used to deliver anticancer agents, with doxorubicin the most frequently incorporated drug, alongside others such as paclitaxel and curcumin. Additionally, co-delivery with microRNAs (miRNAs), including miR-145, miR-200, and miR-139-5p, has been explored to enhance therapeutic outcomes. Current research predominantly relies on subcutaneous xenograft CRC models in mice using the CT26, C26, and HCT116 cell lines. For instance, Sesarman et al. [63] developed liposomal systems capable of co-delivering doxorubicin and curcumin, demonstrating synergistic therapeutic effects. In mice bearing subcutaneous CRC tumours induced by the C26 cell line, this formulation resulted in a substantial reduction in tumour volume, approximately 85% lower than in the control groups, along with decreased angiogenesis and reduced tumour invasiveness [63].

Similarly, miR-200, a microRNA known to suppress tumour metastasis, has been incorporated into nanocarrier systems. Juang et al. [64] reported the co-delivery of miR-200 with irinotecan (IRI) using PEGylated, pH-sensitive, peptide-modified liposomes and solid lipid nanoparticles functionalised with cell-penetrating peptides, tumour vasculature-targeting ligands, and mitochondrial-targeting sequences. Treatment of CT26 tumour-bearing BALB/c mice with these formulations led to significant reductions in tumour volume and systemic toxicity, as evidenced by lower serum levels of biomarkers, including glutamate pyruvate transaminase, creatinine, and creatine kinase-MB [64].

Another approach involved incorporating gallic acid into PEG-PLA-based nanoformulations to address its poor solubility. This system achieved an approximately twofold greater reduction in tumour volume than free gallic acid, along with improved survival rates in CRC xenograft mouse models. Additionally, angiogenesis was reduced by about 90% compared with 75% observed with the free compound [65].

Beyond their use in chemotherapy, nanosystems are also employed to improve radiotherapy. Le Goas et al. [66] created hybrid gold nanoparticles grafted with poly(methacrylic acid) for delivering radioactive iodine (^131^I), resulting in enhanced radiotherapeutic outcomes by promoting greater tumour cell death in mice.

The preclinical literature demonstrates impressive proof-of-concept, but it is uneven in model choice, endpoints, and comparator design. Subcutaneous xenografts dominate, whereas orthotopic and metastatic models that better reproduce the intestinal microenvironment are less common [142]. This creates a major translational gap because nanoparticle distribution is influenced by vascularisation, extracellular matrix, microbiota, immune interactions and route of administration. Natural-product systems illustrate the same problem: curcumin, gallic acid and other phytochemicals may show improved tumour responses after nanoformulation, but the field still needs systematic evidence connecting formulation properties to exposure, pharmacodynamics, and survival [22,63,65]. The next generation of studies should prioritise orthotopic/metastatic models, clinically relevant dosing, pharmacokinetics, toxicity, and direct comparison with standard-of-care treatment.

## 5. Potential Natural Products for the Treatment of Colorectal Cancer

This section focuses on small-molecule natural products that have demonstrated bioactivity against colorectal cancer models and may serve as candidates for further therapeutic development. Most compounds discussed are plant-derived; however, Chaetocochin J is a fungal-derived natural product and is therefore identified separately. The evidence is interpreted critically, with attention to potency, mechanism, nanodelivery status, and the limitations of extrapolating from in vitro activity to therapeutic potential [144].

Mass-concentration IC_50_ values from the earlier draft were not converted where a directly supported molecular-weight basis was not available; they were therefore removed rather than converted using unsupported assumptions. This preserves consistency and traceability to the cited evidence. A clinically relevant candidate must combine reproducible CRC activity with a plausible mechanism, chemical accessibility, acceptable selectivity, manageable ADME properties, and a realistic formulation pathway. On that basis, axidimins C/D are interesting for potency and mechanistic definition but lack direct nanoformulation evidence; berberine has a stronger formulation rationale because poor bioavailability is a recognised limitation; worenine and berbamine have mechanistically informative CRC data but require direct nanodelivery and pharmacokinetic studies; solasonin and nitidine chloride are promising but remain comparatively early in formulation development; and the Melodinus-derived compounds, boldine, venalstonidine, and GB7 acetate require stronger CRC-specific and translational datasets. Chaetocochin J is mechanistically potent but should be considered separately because it is fungal-derived [145,146,147,148,149,150,151,152,153,154,155,156,157,158,159,160]. This comparison identifies where nanotechnology is solving a demonstrated problem and where it is still only a hypothesis.

### 5.1. Axidimins C and D

Axidimins C and D are monoterpenoid indole alkaloid dimers from Melodinus axillaris (Apocynaceae) that show potent cytotoxicity against HCT116 colorectal cancer cells (IC_50_: 5.3 and 3.9 µM) [145]. Their activity is associated with G2/M cell-cycle arrest and apoptosis through the modulation of cyclin B1/cdc2, Bax/Bcl-2, and p38 MAPK signalling [145]. Direct nanoformulations of axidimins have not been established in the literature considered here; therefore, the successful delivery of related indole alkaloids should be regarded as a rationale for future formulation work rather than evidence of an established axidimin nanotherapy.

Since axidimins C and D have defined cell-cycle and apoptotic effects but no established nanoformulation in the evidence base, a logical first study would compare a simple lipid or PLGA formulation with the free compounds for stability, intracellular exposure, and pharmacokinetics before adding active targeting. The key gap is not another proof-of-concept cytotoxicity assay, but demonstration that nanoencapsulation changes exposure sufficiently to improve therapeutic selectivity [13,21,145].

### 5.2. Tabersonine

Tabersonine is a monoterpenoid indole alkaloid associated with Catharanthus/Voacanga species and has demonstrated cytotoxic activity in cancer models, including CRC-relevant investigations [148]. The available evidence supports further investigation, but its molecular targets and the translational value of the reported in vitro activity remain incompletely defined. No direct tabersonine nanoformulation was established in the sources used in this review; consequently, formulation strategies developed for structurally related indole alkaloids should be considered exploratory rather than validated delivery approaches.

Tabersonine should be advanced cautiously because evidence from related indole alkaloids cannot substitute for compound-specific formulation and pharmacology. Structure–formulation relationships, metabolic stability, and direct CRC models should be established before complex targeting is attempted [148].

### 5.3. 19R-Hydroxytabersonine and 11-Hydroxytabersonine

19R-hydroxytabersonine and 11-hydroxytabersonine are monoterpenoid indole alkaloids reported from Melodinus species [149]. Their structural class provides a rationale for investigating antiproliferative activity, but the evidence base for CRC-specific nanodelivery remains limited. The absence of a validated nanoparticle formulation for these compounds is an important translational gap and should be distinguished from evidence obtained with related indole alkaloids.

### 5.4. Chaetocochin J

Chaetocochin J is a fungal-derived epipolythiodioxopiperazine (ETP) alkaloid from the genus Chaetomium and is therefore not a plant-derived natural product [150]. It has demonstrated potent anti-CRC activity in vitro, including an IC_50_ of approximately 0.5 µM, with reported effects involving apoptosis, autophagic cell death, AMPK activation, and suppression of the PI3K/AKT/mTOR axis [151]. Direct nanoparticle formulations of Chaetocochin J remain limited in the literature considered here. Accordingly, related fungal-derived compounds and nanocarriers provide a conceptual basis for future formulation studies, but should not be interpreted as evidence that a Chaetocochin J nanotherapy has been clinically established.

### 5.5. Berberine

Berberine is a protoberberine-type isoquinoline alkaloid found in several medicinal plants. It has demonstrated antiproliferative activity in CRC models, including HCT116 cells, with evidence for effects on telomerase activity and cell-cycle progression [152,153]. Its poor bioavailability makes nanodelivery particularly relevant; however, the benefits of nanoencapsulation should be evaluated against formulation stability, release behaviour, and translational feasibility rather than inferred solely from improved in vitro cytotoxicity.

Berberine is one of the more compelling candidates in this review because its biological activity is accompanied by a recognised bioavailability problem. It is therefore a useful benchmark for testing whether lipid, polymeric, micellar, or hybrid systems provide incremental benefits. Head-to-head studies should compare oral exposure, colonic delivery, tumour concentration, free-drug release, and toxicity rather than reporting only improved cell killing [16,152,153].

### 5.6. Boldine

Boldine is an aporphine alkaloid derived from Peumus boldus. The current reference set does not provide a dedicated CRC study that adequately supports the previously stated numerical IC_50_ value; that value has therefore been removed rather than linked to an unrelated reference. Boldine remains a candidate for further investigation, but its CRC-specific potency and nanodelivery potential require stronger direct evidence.

### 5.7. Worenine

Worenine is an isoquinoline alkaloid derived from Coptis chinensis. It has been reported to inhibit colon cancer cell growth and to interfere with metabolic signalling associated with the Warburg effect through HIF-1α-related mechanisms [154]. Worenine-specific nanodelivery remains underdeveloped, so evidence from related isoquinoline nanocarriers should be considered supportive rather than definitive.

Worenine is mechanistically attractive because its reported effects intersect with tumour metabolism and HIF-1α signalling, but the evidence does not yet establish that nanodelivery improves its therapeutic window. A useful innovation would be to test whether controlled intracellular exposure enhances metabolic pathway modulation while limiting systemic exposure [154].

### 5.8. Venalstonidine

Venalstonidine is a monoterpenoid indole alkaloid associated with Melodinus species [155]. CRC-specific cytotoxicity data remain limited in the reference set used here. Accordingly, broad statements about the activity of the MIA class should not be interpreted as direct evidence of venalstonidine efficacy, and the absence of a validated venalstonidine-specific nanoparticle formulation remains a significant gap.

### 5.9. Solasonin

Solasonin is a steroidal glycoalkaloid isolated from Solanum nigrum. It has demonstrated activity in cancer models, with CRC-relevant effects involving histone deacetylase-associated signalling and p53-related pathways [156]. The evidence supports further investigation of solasonin as a candidate natural product, but direct evidence for a solasonin-specific nanocarrier remains limited.

Solasonin has a defined CRC-relevant signalling rationale, but direct formulation evidence remains limited. Its steroidal glycoalkaloid chemistry makes comparative solubility, membrane interaction, and toxicity studies especially important before selecting a carrier [156].

### 5.10. Berbamine

Berbamine is a bisbenzylisoquinoline alkaloid found in Berberis species. Earlier work supports its broader pharmacological activity [157]. Recent CRC evidence indicates that berbamine can suppress tumour-cell proliferation and signalling through receptor tyrosine kinase/Akt-associated pathways [158]. Berbamine-specific nanodelivery remains insufficiently developed, highlighting the need for formulation studies that establish pharmacokinetics, tumour exposure, and safety.

Berbamine is supported by CRC-specific signalling data, yet its nanodelivery remains underdeveloped. Future work should establish whether a carrier improves tumour exposure without masking the pharmacology or increasing off-target toxicity; this should be tested against free berbamine and a simple carrier control [157,158].

### 5.11. GB7 Acetate

GB7 acetate is a galbulimima alkaloid derived from Galbulimima belgraveana. It has demonstrated anticancer activity in CRC cells, including effects on proliferation and colony formation [159]. The compound is therefore a potential lead for further development, although direct GB7 acetate nanoformulations are not established in the reference set. Any proposed nanodelivery strategy should be supported by comparative pharmacokinetic and toxicity data.

GB7 acetate is an example of a compound for which biological activity is ahead of formulation science. Rather than assuming that nanoencapsulation will improve efficacy, future studies should first define solubility, stability, metabolism, and therapeutic window, then select a carrier accordingly [159].

### 5.12. Nitidine Chloride

Nitidine chloride is a benzophenanthridine alkaloid derived from Zanthoxylum nitidum. It has been reported to inhibit proliferation and induce apoptosis in CRC cells through p53-associated mechanisms [160]. These findings support its consideration as a candidate for nano-enabled delivery, but direct formulation and in vivo translational evidence remain necessary before therapeutic conclusions can be drawn. Nitidine chloride has a plausible apoptosis-related mechanism and a strong rationale for nano-enabled delivery, but direct in vivo formulation evidence remains necessary. A useful development pathway would compare polymeric, lipid, and hybrid carriers for tumour exposure and systemic toxicity before introducing receptor-targeting ligands [160,161].

## 6. Additional Nanoparticle Delivery Systems and Integration Approaches for Therapeutic Interventions Using Small Natural Products

### 6.1. Solid Lipid Nanoparticles (SLNs)

Solid lipid nanoparticles (SLNs) are colloidal delivery systems composed of biocompatible solid lipids and stabilised with surfactants, designed to improve the delivery of poorly soluble plant-derived alkaloids in colorectal cancer (CRC) therapy. They are particularly advantageous for compounds such as berberine, nitidine chloride, and solasonine, which are often limited by low oral bioavailability and rapid elimination. The solid lipid core of SLNs enhances the physicochemical stability of encapsulated compounds, protects them from enzymatic degradation, and supports sustained, controlled drug release [21].

SLNs are attractive where the principal problem is poor solubility or chemical instability, but their lipid matrix can also constrain loading and release. For berberine, nitidine chloride, and solasonin, the important question is whether improved exposure is maintained after oral administration and whether the formulation survives gastrointestinal conditions. These systems should be compared with nanostructured lipid carriers, liposomes, and polymeric particles before being advanced as the preferred platform [21,162].

In CRC-related research, SLNs have demonstrated improved cellular uptake in cancer cell lines such as HCT116 and SW480 [159], resulting in greater cytotoxicity than non-encapsulated drugs. Their nanoscale size enables passive accumulation in tumour tissues via the enhanced permeability and retention (EPR) effect. Their lipid-based composition improves biocompatibility and reduces systemic toxicity. Moreover, SLNs can be customised with surface modifications, such as PEGylation or the attachment of targeting ligands, to increase tumour specificity. Overall, these qualities make SLNs a promising platform for enhancing the therapeutic effectiveness of natural alkaloids in CRC treatment.

### 6.2. Polymeric Micelles

Polymeric micelles are nanoscale assemblies that spontaneously form from amphiphilic block copolymers, creating a core–shell structure that can encapsulate hydrophobic bioactive compounds in the inner core. This property markedly enhances the solubility and stability of poorly water-soluble plant-derived alkaloids [13]. These systems are particularly suitable for compounds such as berberine, worenine, venalstonidine, and camptothecin derivatives, which are commonly investigated in colorectal cancer therapy.

Polymeric micelles are particularly logical for hydrophobic natural products, but their apparent advantages can be weakened by dilution-induced destabilisation and premature drug release after administration. For berberine or related alkaloids, future work should therefore measure micelle integrity in biological fluids and quantify free versus micellar drugs during pharmacokinetic studies rather than assuming that improved solubilisation equals improved tumour delivery [13,47].

These nanocarriers facilitate greater drug buildup at tumour sites mainly via the enhanced permeability and retention (EPR) effect. Additionally, polymeric micelles are engineered for controlled, stimuli-responsive drug release, often reacting to the acidic conditions typical of tumour microenvironments. This approach enhances both therapeutic effectiveness and targeted delivery [47].

In colorectal cancer cell lines such as HCT116 and HT29, micelle-formulated alkaloids have shown increased intracellular uptake and enhanced apoptosis compared with free compounds, as discussed in the studies [162]. Polymeric micelles also reduce premature drug degradation and improve pharmacokinetic profiles, making them suitable for systemic administration. Their core–shell structure enables the co-loading of multiple agents, supporting combination therapy approaches such as pairing alkaloids with chemotherapy or pathway inhibitors. This makes polymeric micelles a versatile platform for optimising plant-derived anticancer agents in colorectal cancer treatment strategies [47].

### 6.3. Gold and Metallic Nanoparticles

Gold nanoparticles (AuNPs) and other metallic nanocarriers have attracted attention for colorectal cancer therapy because of their distinctive physicochemical and optical properties [55]. These systems can be functionalised with plant-derived alkaloids, such as nitidine chloride, berbamine, and berberine, enabling targeted delivery and enhanced cytotoxicity [62]. AuNPs are particularly valuable for image-guided therapy and theranostic applications, as they can be tracked using imaging modalities while delivering therapeutic payloads [22]. In CRC cell models such as HCT116 and RKO, alkaloid-conjugated metallic nanoparticles have shown improved induction of apoptosis and stronger inhibition of proliferation compared with free drugs. Their surface plasmon resonance properties can also enhance localised heat generation under irradiation, supporting photothermal therapy combined with chemotherapy. Additionally, metallic nanoparticles allow for precise surface engineering with antibodies or peptides for tumour-specific targeting. However, concerns about long-term biocompatibility and clearance remain important considerations in their translational development for colorectal cancer treatment [22].

### 6.4. Mesoporous Silica Nanoparticles (MSNs)

Mesoporous silica nanoparticles (MSNs) are highly porous nanocarriers known for their large surface area, tunable pore size, and high capacity to load plant-derived alkaloids used in colorectal cancer treatment [116]. They are particularly effective for hydrophobic compounds such as berberine, solasonine, and worenine, which benefit from controlled, sustained release. MSNs enable pH-responsive and enzyme-triggered drug release, making them well-suited to the acidic tumour microenvironment of colorectal cancer. In vitro studies with CRC cell lines such as HCT116 and SW620 have shown improved cytotoxicity and higher rates of apoptosis when alkaloids are delivered via silica-based systems compared with free drugs. Their rigid structure protects drugs from premature degradation, and surface functionalisation enables targeted delivery via ligands or antibodies. Additionally, MSNs enable the co-delivery of multiple therapeutics, supporting synergistic approaches that combine natural alkaloids with chemotherapy or gene therapy. These features make mesoporous silica nanoparticles a versatile platform for enhancing anticancer treatments [116].

MSNs offer an unusually flexible loading-and-gating architecture, making them attractive for natural products that require high loading or protected release. However, the field needs more evidence on biodegradation, clearance, and comparative benefit versus biodegradable organic carriers. The most useful next studies would combine quantitative release, pharmacokinetics, and tissue distribution with CRC efficacy rather than relying on cell-line potency [47,116].

### 6.5. Dendrimers

Dendrimers are highly branched, nanoscale polymers with a well-defined structure that enable precise control over drug loading and surface modifications [56]. They should be increasingly studied for delivering plant-derived alkaloids such as berberine, nitidine chloride, and berbamine in colorectal cancer treatment. Their multivalent surface enables high drug conjugation and targeted delivery via ligand attachment, improving selectivity for tumour cells. In CRC models such as HCT116 and SW480, dendrimer-based formulations may improve cellular uptake and intracellular delivery when appropriately designed, but these benefits require direct experimental validation in CRC models [163]. Their internal cavities can encapsulate hydrophobic molecules, and adjusting the surface charge enhances interaction with cancer cell membranes. Dendrimers also support controlled release and reduce systemic toxicity, overcoming common issues with traditional alkaloid therapy. Additionally, they can be engineered for the co-delivery of genes and drugs, allowing combined targeting of oncogenic pathways and chemotherapy. However, clinical use remains limited due to manufacturing challenges and potential toxicity at higher generations [98].

Dendrimers may be particularly valuable when multivalent targeting or co-delivery is genuinely required, but they should not be selected merely because their surface is highly functionalised. For berberine, nitidine chloride, or berbamine, the burden of proof should include generation-dependent toxicity, biodistribution, clearance, and manufacturing reproducibility [35,37,56,98].

### 6.6. Exosome-Based Delivery Systems

Exosome-based drug delivery systems use naturally occurring extracellular vesicles to transport plant-derived alkaloids, offering high biocompatibility and intrinsic targeting. These systems are particularly promising for colorectal cancer therapy with compounds such as berberine, nitidine chloride, and solasonine, owing to their ability to cross biological barriers and preferentially accumulate in tumour tissue [23]. Exosomes derived from cancer or stem cells can be engineered to carry alkaloids and deliver them directly to HCT116, HT29, and SW480 colorectal cancer cells, thereby enhancing uptake and cytotoxicity. Their lipid bilayer protects the drug cargo from enzymatic degradation and immune clearance, improving systemic stability. Additionally, exosomes naturally express surface proteins that facilitate cell-specific recognition and uptake, reducing off-target effects. Preclinical studies suggest that exosome-encapsulated alkaloids induce apoptosis and suppress tumour growth more effectively than free drugs. However, scalability and standardisation remain key challenges for clinical translation [164].

Exosome and extracellular-vesicle systems represent a major conceptual shift from synthetic carriers toward biomimetic delivery, including plant-derived extracellular vesicles [23,164]. Their promise is tempered by the heterogeneity of the source material, loading variability, purification, potency assays, and scale-up. The innovation opportunity is to establish reproducible, well-characterised vesicle products and determine whether their biological advantages remain after standardised manufacture.

### 6.7. Hybrid Lipid–Polymer Nanoparticles

Hybrid lipid–polymer nanoparticles combine the structural stability of polymeric cores with the biocompatibility of lipid shells, providing an efficient platform for delivering plant-derived alkaloids in colorectal cancer therapy [161]. These systems are particularly suitable for compounds such as berberine, worenine, and nitidine chloride, which benefit from both controlled release and enhanced membrane interaction. The polymer core, often composed of PLGA, ensures sustained drug release and structural integrity, while the lipid outer layer improves cellular uptake and mimics biological membranes. In CRC models such as HCT116 and SW480, hybrid nanoparticles have demonstrated superior cytotoxicity, enhanced apoptosis, and greater intracellular accumulation than single-component systems [165]. Their dual nature enables multifunctional modification, including PEGylation and ligand targeting, to improve tumour specificity. These systems also support the co-delivery of multiple therapeutic agents, enabling synergistic anticancer strategies. Their combined properties make hybrid nanoparticles a promising platform, but comparative in vivo evidence is still needed to establish whether their added complexity produces a clinically meaningful advantage [166].

Hybrid systems are attractive because they combine polymeric structural control with lipid-mediated membrane interactions, but the added layers also increase process complexity. Their value should therefore be demonstrated through head-to-head studies against the best-performing single-component carrier for the same natural product, using efficacy, pharmacokinetics, toxicity, and manufacturing robustness as co-primary development criteria [161].

## 7. Integration Approaches for Therapeutic Interventions Using Plant-Derived Anti-Cancer Compounds and Delivery Systems for the Treatment of Colorectal Cancer

Targeting should be considered the final layer of a development sequence, not the starting point. Across 2010–2026, ligand engineering has become increasingly sophisticated, but the evidence remains strongest when the ligand is benchmarked against an otherwise identical non-targeted carrier [15,16,23,24,25,26,27,28,29,30,31,32,33]. For the natural products reviewed here, the first objective should be to establish adequate exposure and controlled release; the second is to demonstrate that a ligand improves cell-specific delivery; and only then should dual targeting or multimodal functions be considered. This hierarchy can prevent over-engineering and make the contribution of each design element experimentally testable. Targeted delivery of plant-derived alkaloids, such as berberine, nitidine chloride, berbamine, solasonine, worenine, boldine, and GB7 acetate, can be significantly improved through the surface functionalisation of nanocarriers [167]. Across nanoparticle platforms, including PLGA nanoparticles, lipid nanoparticles, solid lipid nanoparticles, polymeric micelles, mesoporous silica nanoparticles, dendrimers, gold nanoparticles, exosomes, and hybrid lipid–polymer systems, ligand engineering enables the selective recognition of colorectal cancer cells, particularly in models such as HCT116, HT29, SW480, and SW620. These targeting strategies enhance receptor-mediated uptake, improve tumour accumulation via the EPR effect, and reduce off-target toxicity. Functional ligands can be grouped by receptor class or biological target, enabling the rational design of nanomedicines tailored to the molecular profiles of colorectal cancer [168].

### 7.1. Folic Acid (Folate Receptor Targeting)

Folic acid is a widely used targeting ligand because of folate receptor overexpression in many colorectal cancer cells, including HCT116 and HT29. Nanoparticles such as PLGA systems, lipid nanoparticles, and mesoporous silica nanoparticles loaded with alkaloids such as berberine and nitidine chloride can be functionalised with folate to enhance receptor-mediated endocytosis. This approach increases intracellular drug accumulation and promotes apoptosis by raising the local drug concentration. Folate-targeted systems have shown improved cytotoxicity and reduced systemic exposure in vitro. The ligand is typically conjugated to PEG chains on nanoparticle surfaces, enabling stable circulation and selective tumour binding. This strategy is particularly effective for hydrophobic alkaloids that require improved solubility and uptake [24,25].

Folate targeting is attractive, but receptor expression is heterogeneous and not synonymous with selective tumour delivery. Future studies should quantify receptor expression in the exact CRC model, compare targeted versus non-targeted carriers, and determine whether the ligand improves tumour-to-normal exposure rather than only cellular uptake [24,25].

### 7.2. Peptides (RGD, T7, and Targeting Motifs)

Peptide ligands such as RGD (arginine–glycine–aspartate) and T7 peptides are commonly used to target integrins (especially αvβ3) and transferrin receptors, which are overexpressed in colorectal cancer [26]. These ligands can be attached to polymeric micelles, dendrimers, gold nanoparticles, and hybrid lipid–polymer nanoparticles carrying alkaloids such as berbamine, worenine, and solasonine. RGD-modified nanoparticles enhance tumour cell adhesion and internalisation, thereby improving drug delivery to HCT116 and SW480 cells. T7 peptides facilitate transferrin receptor-mediated uptake, which is associated with rapidly proliferating cancer cells. These peptide-functionalised systems enhance apoptosis, inhibit proliferation, and improve selectivity compared with non-targeted formulations. Peptide conjugation is typically achieved through covalent bonding to surface functional groups, enabling stable and highly specific tumour targeting [27].

Peptide ligands permit flexible targeting but can be degraded, masked by protein corona formation or lose efficacy when receptor expression varies. Multi-ligand systems should therefore be evaluated against single-ligand controls to establish whether additional targeting complexity genuinely improves penetration or therapeutic index [26,27,33].

### 7.3. Antibodies and Antibody Fragments (EGFR, CEA Targeting)

Monoclonal antibodies and antibody fragments are highly specific ligands that target overexpressed colorectal cancer markers, such as epidermal growth factor receptor (EGFR) and carcinoembryonic antigen [28]. These ligands are particularly suitable for gold nanoparticles, exosomes, and dendrimers, which can be loaded with alkaloids such as nitidine chloride, berbamine, and berberine [29]. EGFR-targeted systems enhance uptake in cancer cells, leading to increased apoptosis and the inhibition of proliferation pathways such as PI3K/AKT/mTOR, which are also affected by compounds like worenine. Antibody functionalization enables highly selective binding and receptor-mediated internalisation, improving therapeutic index. These systems are often engineered using PEG linkers to maintain antibody orientation and stability on the nanoparticle surface [30].

Antibody-based targeting can provide high specificity, but it also increases molecular size, manufacturing complexity and potential immunological or pharmacokinetic liabilities. For natural-product nanomedicines, the development question is whether antibody-mediated internalisation produces sufficient benefit to justify this complexity compared with peptide or aptamer targeting [28,29].

### 7.4. Aptamers (Nucleic Acid-Based Targeting)

Aptamers are short single-stranded nucleic acids that bind specifically to tumour-associated proteins, offering an alternative to antibodies with lower immunogenicity. They can be conjugated to mesoporous silica nanoparticles, PLGA nanoparticles, and polymeric micelles carrying alkaloids such as berberine, solasonine, and boldine. Aptamers targeting markers such as nucleolin or EpCAM enhance uptake in colorectal cancer cells, including HCT116 and SW620, improving cytotoxic efficacy and apoptosis induction. Their small size allows for deeper tumour penetration and efficient receptor binding. Aptamer-functionalized nanoparticles also support controlled release and enhanced intracellular delivery, improving the pharmacological activity of poorly soluble alkaloids [147].

Aptamers may offer a useful compromise between antibody specificity and smaller carrier architecture, but stability, batch consistency and performance in biological fluids must be established. Direct comparisons with antibodies and peptides using the same natural-product payload would clarify where aptamers provide a genuine advantage [147].

### 7.5. Sugar Ligands (Mannose and Glucose Transport Targeting)

Sugar-based ligands such as mannose and glucose derivatives exploit the high metabolic demand of colorectal cancer cells, which often overexpress glucose transporters (GLUT1) and lectin receptors [30]. These ligands are commonly used to conjugate to lipid nanoparticles, solid lipid nanoparticles, and polymeric micelles that can be designed to carry alkaloids such as berberine and GB7 acetate. Glucose-functionalized systems enhance uptake through transporter-mediated endocytosis, increasing intracellular drug concentration in HCT116 cells. This strategy is particularly effective for compounds targeting metabolic pathways, such as GB7 acetate, which acts via AMPK signalling. Sugar-functionalized nanoparticles improve tumour selectivity, reduce systemic toxicity, and enhance metabolic disruption in cancer cells [31].

Metabolic targeting is conceptually attractive for compounds that themselves modulate tumour metabolism, but increased uptake through glucose-related pathways could also affect normal metabolically active tissues. Future work should therefore quantify tumour selectivity and systemic exposure rather than assuming that increased uptake is inherently beneficial [30,31].

### 7.6. Exosome Surface Engineering (Biomimetic Targeting)

Surface modification of exosomes via ligand conjugation, including peptides and antibodies, serves to refine their biodistribution and minimize off-target effects [32]. These systems can be potentially used to carry plant-derived alkaloids, including berberine, solasonine, and nitidine chloride, enabling natural membrane compatibility and enhanced tumour uptake. Exosomes derived from cancer cells can be modified to express surface proteins that preferentially bind HCT116, HT29, and SW480 cells, thereby improving intracellular delivery and inducing apoptosis. Unlike synthetic nanoparticles, exosomes inherently possess membrane proteins that support immune evasion and prolonged circulation. Functionalization further enhances their ability to deliver cytotoxic alkaloids directly to colorectal tumour sites, improving therapeutic efficiency [23].

Exosome engineering could combine biomimicry with ligand targeting, but the field still lacks universally standardised manufacturing and potency criteria. For plant-derived alkaloids, the key unanswered question is whether engineered vesicles improve delivery sufficiently to compensate for production complexity and heterogeneity [23,32].

### 7.7. Integrated Targeting and Multimodal Functionalization

Advanced nanoparticle systems increasingly combine multiple ligands to achieve dual or triple targeting strategies, such as folate–RGD or antibody–peptide combinations [33]. These are applied to PLGA nanoparticles, hybrid lipid–polymer systems, dendrimers, and mesoporous silica nanoparticles loaded with alkaloids like berbamine, nitidine chloride, and worenine. Such integration enhances selectivity for colorectal cancer cells while simultaneously targeting multiple receptors (e.g., EGFR and integrins). This multimodal approach improves tumour penetration, reduces resistance mechanisms, and enhances apoptosis across CRC models, including HCT116 and SW480. Combined targeting also allows for the synergistic delivery of alkaloids with chemotherapeutic agents, further increasing anticancer efficacy and supporting precision nanomedicine strategies in colorectal cancer therapy [34].

Dual and triple-targeting systems represent the logical endpoint of the 2010–2026 trend toward multifunctionality, but they also create the greatest risk of over-engineering. The field needs ablation-style studies in which each ligand or function is removed sequentially to determine its individual contribution. For natural-product CRC therapy, the most compelling future systems will be those in which each added function—targeting, responsive release, imaging or co-delivery—has a measurable and mechanistically justified benefit [33,34].

## 8. Conclusions and Future Perspectives

The field has evolved from relatively simple encapsulation and circulation-extension strategies toward precision, responsive, biomimetic, and multimodal nanomedicines. Yet the accumulated literature does not demonstrate that increasing nanosystem complexity automatically increases clinical value. The most defensible interpretation is that nanotechnology is useful when it corrects a clearly defined limitation of a particular payload and when the resulting formulation is demonstrably better than appropriate simpler controls [11,14,16,21,22,35,36,37,38,39,53]. Recent advances in nanomedicine indicate that the value of a nanocarrier for CRC therapy depends on the interaction between carrier architecture, payload properties, biological targeting, pharmacokinetics, and translational feasibility rather than on nanoparticle size alone. Liposomes, polymeric systems, dendrimers, metallic and magnetic nanoparticles, mesoporous silica, solid lipid nanoparticles, micelles, exosomes, and hybrid platforms each provide distinct advantages, but also introduce platform-specific limitations involving stability, toxicity, clearance, manufacturing, and regulatory approval [13,14,22,35,36,37,38,39,46,47,55,56,62,116].

Plant-derived natural products provide a complementary source of chemically diverse anticancer candidates, while fungal-derived compounds such as Chaetocochin J should be distinguished from plant-derived compounds. Across the compounds reviewed, the principal development barriers include poor solubility, rapid metabolism, limited bioavailability, insufficient tumour selectivity, and limited direct evidence for clinically relevant nanodelivery. The strongest future opportunities therefore lie in integrating natural-product pharmacology with rational carrier design, responsive release, biomimetic delivery, and validated targeting strategies [16,23,24,25,26,27,28,29,161]. Future studies should prioritise rigorous in vivo pharmacokinetic and biodistribution studies, dose–response relationships, long-term toxicity, manufacturing reproducibility, and well-designed translational studies. Multifunctional and stimuli-responsive systems are promising, but added complexity should be justified by demonstrable therapeutic benefit. A clinically useful nanomedicine must ultimately achieve a favourable balance between efficacy, safety, manufacturability, stability, and regulatory acceptability. Thus, nanotechnology combined with carefully selected natural products remains a promising, but still predominantly preclinical, strategy for precision CRC therapy.

First, natural products should be triaged by pharmacological need: compounds with poor solubility may favour lipid or micellar systems; compounds with rapid degradation may benefit from protective polymeric or lipid matrices; locally acting compounds may be better suited to oral/chitosan or colon-responsive delivery; and compounds requiring intracellular precision may justify ligand-functionalised or biomimetic systems. Second, head-to-head comparisons using the same payload and CRC model are needed to distinguish genuine platform advantages from formulation-specific effects. Third, future studies should progress beyond subcutaneous xenografts toward orthotopic, metastatic, immune-competent and advanced human-relevant models, with integrated pharmacokinetic, biodistribution, pharmacodynamic, and toxicity endpoints [11,14,16,41,53]. Fourth, manufacturing reproducibility, scale-up, long-term stability, and regulatory characterisation should be treated as design constraints from the beginning rather than late-stage obstacles. Finally, the plant-derived candidates identified here, particularly berberine, berbamine, nitidine chloride, solasonin, worenine, and the Melodinus alkaloids represent a useful innovation space precisely because many remain under-formulated. The major opportunity is not to create the most complex nanoparticle, but to create the simplest clinically credible system that measurably improves exposure, selectivity, and therapeutic index.

## Figures and Tables

**Table 1:** Advantages and disadvantages of existing nanoformulations.

Nanosystem	Advantage	Disadvantage	References
Nanoemulsion	It can be used instead of liposomes and micelles. Improves bioavailability of the drug. Non-irritant and non-toxic. Excellent absorption.	The surfactant must not be toxic. Stability is chiefly influenced by environmental conditions such as pH and temperature. High concentrations of co-surfactant and surfactant are critical for sustaining nanodrops.	[45]
Dendrimers	Lower polydispersity index. The outer surface of dendrimers has multiple active functional groups. They can be synthesized and designed for specific applications such as CRC. It can be used to deliver DNA and proteins.	Insufficient loading capacity, drug expulsion after polymorphic transition during storage.	[46]
Liposomes	Increased efficacy and therapeutic index of the drugs and stability via encapsulation. Non-toxic, biodegradable, biocompatible, non-immunogenic for systemic and non-systemic administrations. Decreased exposure of sensitive tissues to toxic drugs.	Low solubility, short half-life and leakage and fusion of the encapsulated drug. High cost and not stable.	[21]
Polymers	Stability of any volatile pharmaceutical agents. Delivers a high concentration of pharmaceutical agents to the desired location. It can easily be merged into other systems associated with drug delivery.	Noxious factors such as toxicity, reactive residues, unreacted monomers, the risk of a chemical reaction, and unwanted oligomer formation.	[13]
Silica	Their properties make them attractive and suitable for in vivo based studies. They are non-toxic, biodegradable, biocompatible, and versatile.	Expensive. The skeletal stability of silica is very low, and is further reduced by sheer fluid stress, which can happen during circulation.	[47]

**Table 3:** Selected nanosystems investigated in preclinical colorectal cancer models.

Nanosystem	Application	Transported Molecule	Animal Model	Active Targeting	References
PEGylated hyaluronic acid NPs	Treatment	Doxorubicin	SD rats and CT26 subcutaneous tumor-bearing BALB/c mice subcutaneously	Hyaluronic acid induces CD44+ targeting tumors	[67]
Polymersomes	Treatment	Doxorubicin	BALB/c mice and HCT-116 subcutaneous tumor-bearing BALB/c nude mice	Tfr-binding peptide	[68]
NaYF_4_:Yb/Tm upconversion NPs, SiO_2_-coated and with PEI	Treatment	miR-145 vector	Colon cancer mice model	Hyaluronic acid	[143]
PLGA NPs	Treatment	Paclitaxel	LS174T subcutaneous tumor-bearing BALB/c (nu/nu) mice	Tumor-penetrating peptide iRGD co-administration	[46,69]
PEG-pH-sensitive/peptide modified liposomes and solid lipid NPs	Treatment	Irinotecan and miR-200	CT26 subcutaneous tumor-bearing BALB/c mice	Cell-penetrating peptide, the ligand to new tumor blood vessels, and mitochondria-targeting peptide	[64]
Hybrid poly(methacrylic acid)-grafted gold NPs	Treatment (RT)	^131^I	B16-NIS subcutaneous tumor-bearing BALB/c nude mice	-	[66]
Cationic liposome-based NPs	Treatment	miR-139-5p	HCT8 subcutaneous tumor-bearing BALB/c nude mice and HCT116-luc peritoneal xenograft in BALB/c mice	EpCAM and Apt	[70]
α-Phase erbium-based rare-earth NPs	Imaging	-	CT26 subcutaneous tumor-bearing BALB/c mice	Anti-PD-L1 mAb	[71]
Silica-gold nanoshells	PTT, HT	-	CT26 subcutaneous tumor-bearing BALB/c mice	-	[72]
PEG-PLGA NPs	Treatment	Sorafenib and PEDF	C26 subcutaneous tumor-bearing BALB/c mice	-	[73]
Nanocapsules	Treatment	SN38 and regorafenib	CT26 subcutaneous tumor-bearing BALB/c mice	-	[74]
Liposomal NPs	Treatment	Moeixitecan (SN38 prodrug)	HT29 subcutaneous tumor-bearing BALB/c nude mice	-	-
Poly(amino acid) nanoparticles	Treatment	Evodiamine	ICR mice and LoVo subcutaneous tumor-bearing BALB/c nude mice	EGFR	[75]
Layer-by-layer assembly PLA NPs	Treatment (chemotherapy and radiation)	Raltitrexed	CT26 subcutaneous tumor-bearing BALB/c mice	Hyaluronic acid	[76]
Poly(ethylene oxide)-poly(butylene oxide) (PEO-PBO) diblock co-polymer	Treatment	Linoleic acid and SN38	Sprague Dawley rats, ICR mice, and HCT116 subcutaneous tumor-bearing BALB/c mice	Linoleic acid	[77]
Liposome-protamine lipoplex	Treatment	In vitro transcribed mRNA-T34A	C26 subcutaneous tumor-bearing BALB/c mice, and intravenous and intraperitoneal injection for metastatic model	-	[128]
PEGylated hollow mesoporous ruthenium NPs	Treatment and imaging	RBT (fluorescent probe with antitumor activity)	CT26-CEA subcutaneous tumor-bearing BALB/c mice	SS-Fc, antibodies for CD16 and CEA	[90]
Minicapsules that encapsulate chitosan NPs	Treatment	7-hydroxy-staurosporine (to activate caspase 3)	Caco 2 cell inoculated in the colon mucosa of C57BL/6 mice	Folate	[111]
PEG-PLGA NPs with TPP	Treatment	HCPT	SW620 subcutaneous tumor-bearing nude mice	Folate	[78]

*Tfr* transferrin, *PEI* polyetherimide, *RT* radiotherapy, *EpCAM* epithelial cell adhesion molecule, *Apt* aptamer, *anti-PD-L1* programmed cell death-1 ligand-1 antibody, *PTT* photothermal therapy, *HT* hyperthermia, *PEDF* pigment epithelium-derived factor, *HCPT* 10-hydroxycamptothecin, *TPP* triphenylphosphonium, *SPIONs* superparamagnetic iron oxide nanoparticles and (−) accumulate passively in the tumor.

## Data Availability

No new data were created or analyzed in this study. Data sharing is not applicable to this article.

## Abbreviations

The following abbreviations are used in this manuscript:

AgNPs	Silver Nanoparticles
AKT	Protein Kinase B
AMPK	AMP-Activated Protein Kinase
API/APIs	Active Pharmaceutical Ingredient(s)
Apt	Aptamer
AuNPs	Gold Nanoparticles
CEA	Carcinoembryonic Antigen
CNPs	Chitosan Nanoparticles
CNTs	Carbon Nanotubes
CRC	Colorectal Cancer
DDS	Drug Delivery System
DNA	Deoxyribonucleic Acid
DOX	Doxorubicin
DS	Delivery System
EGF	Epidermal Growth Factor
EGFR	Epidermal Growth Factor Receptor
EpCAM	Epithelial Cell Adhesion Molecule
EPE	Enhanced Permeability Effect
EPR	Enhanced Permeability and Retention
ETP	Epipolythiodioxopiperazine
FDA	Food and Drug Administration
FdUMP	Fluorodeoxyuridine Monophosphate
FdUTP	Fluorodeoxyuridine Triphosphate
FGFR	Fibroblast Growth Factor Receptor
FITC	Fluorescein Isothiocyanate
FUTP	Fluorouridine Triphosphate
GQDs	Graphene Quantum Dots
HCPT	10-Hydroxycamptothecin
HDAC	Histone Deacetylase
HIF-1α	Hypoxia-Inducible Factor 1-alpha
HSA	Human Serum Albumin
HT	Hyperthermia
IC_50_	Half Maximal Inhibitory Concentration
iRGD (or RDG)	Internalizing Arginine–Glycine–Aspartic peptide
IV	Intravenous
JAK/STAT	Janus Kinase/Signal Transducer and Activator of Transcription
LPs	Liposomes
MAPK	Mitogen-Activated Protein Kinase
MIA	Monoterpenoid Indole Alkaloid
miRNA/miR	MicroRNA
MNPs	Metallic Nanoparticles
MSNs	Mesoporous Silica Nanomaterials
mTOR	Mechanistic Target of Rapamycin
MWCNTs	Multi-Walled Carbon Nanotubes
NDDS	Nano-Drug Delivery System(s)
NE	Nanoemulsion
NIR	Near Infrared
NLs	Nanoliposomes
NP/NPs	Nanoparticle(s)
NS/NSs	Nanosystem(s)
NVs	Nanovectors
PAMAM	Polyamidoamine
PCL	Polycaprolactone
PDGFRα	Platelet-Derived Growth Factor Receptor Alpha
PDT	Photodynamic Therapy
PEDF	Pigment Epithelium-Derived Factor
PEG	Polyethylene Glycol
PI3K	Phosphoinositide 3-Kinase
PLGA	Poly(lactic-co-glycolic acid)
PNPs	Polymeric Nanoparticles
PPI	Poly(propylene-imine)
PTT	Photothermal Therapy
PTX	Paclitaxel
QDs	Quantum Dots
RES	Reticuloendothelial System
RFA	Radiofrequency Ablation
RNA	Ribonucleic Acid
ROS	Reactive Oxygen Species
RT	Radiotherapy
scFv	Single-chain Variable Fragment
SLNs	Solid Lipid Nanoparticles
SPIONs	Superparamagnetic Iron Oxide Nanoparticles
SWCNTs	Single-Walled Carbon Nanotubes
TPP	Triphenylphosphonium
VEC	Vascular Endothelial Cell
VEGF	Vascular Endothelial Growth Factor
